# Di-Lineage Hepatic Spheroids From Human Donors Capture Steatotic Liver Disease

**DOI:** 10.1016/j.jcmgh.2026.101804

**Published:** 2026-05-08

**Authors:** Tanmoy Dutta, Lohitesh Kovooru, Annika Thorsell, Carmelo Pujia, Samantha Maurotti, Kavitha Sasidharan, Rosellina M. Mancina, Stefano Romeo

**Affiliations:** Department of Molecular and Clinical Medicine, Institute of Medicine University of Gothenburg, Gothenburg, Sweden; Department of Molecular and Clinical Medicine, Institute of Medicine University of Gothenburg, Gothenburg, Sweden; Department of Medicine (H7), Centre for Reproduction, Metabolism, and Molecular medicine (CeRM), Karolinska Institute, Huddinge, Sweden; Proteomics Core Facility, BioMS, SciLifeLab, University of Gothenburg, Gothenburg, Sweden; Department of Medical and Surgical Sciences, University Magna Graecia, Catanzaro, Italy; Ribocure Pharmaceuticals AB Gothenburg, Gothenburg, Sweden; Department of Molecular and Clinical Medicine, Institute of Medicine University of Gothenburg, Gothenburg, Sweden; Department of Medicine (H7), Centre for Reproduction, Metabolism, and Molecular medicine (CeRM), Karolinska Institute, Huddinge, Sweden; Department of Life Science, Health, and Health Professions, Link Campus University, Rome, Italy; Research Unit of Clinical Medicine and Hepatology, Department of Medicine and Surgery, Università Campus Bio-Medico di Roma, Rome, Italy; Department of Molecular and Clinical Medicine, Institute of Medicine University of Gothenburg, Gothenburg, Sweden; Department of Medicine (H7), Centre for Reproduction, Metabolism, and Molecular medicine (CeRM), Karolinska Institute, Huddinge, Sweden; Department of Medical and Surgical Sciences, University Magna Graecia, Catanzaro, Italy; Department of Cardiology, Sahlgrenska University Hospital, Gothenburg, Sweden; Department of Endocrinology, Karolinska University Hospital, Huddinge, Sweden

**Figure:**
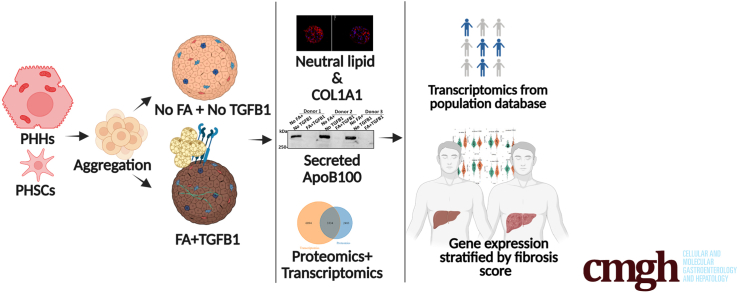

Metabolic dysfunction-associated steatotic liver disease (MASLD) is a prevalent chronic liver condition affecting approximately 32% of the global population.^1^ MASLD encompasses a spectrum of liver conditions, ranging from hepatic steatosis to inflammation, steatohepatitis, fibrosis, ultimately leading to cirrhosis and hepatocellular carcinoma.^2^^,^^3^ Despite its clinical burden, the molecular mechanisms driving the transition from steatosis to metabolic dysfunction-associated steatohepatitis remain poorly understood, hindering the development of reliable biomarkers and effective therapies.

Traditional models, including immortalized cell lines and induced pluripotent stem cell–derived organoids, offer valuable insights but have limitations.^4^^,^^5^ Spheroids from immortalized cell lines fail to mimic physiological conditions, whereas induced pluripotent stem cell–derived organoids require validation to confirm successful differentiation and appropriate cell type proportions. These limitations underscore the need for advanced 3-dimensional in vitro models bridging between basic research and clinical applications.^6^

To address these shortcomings, we utilized di-lineage primary human spheroids consisting of primary human hepatocytes (PHHs) and primary human hepatic stellate cells (PHSCs) at their physiological ratio (24:1). These spheroids were cultured in regular media or fibrosis-inducing condition using free fatty acids and transforming growth factor β 1 (TGFB1), a known promoter of fibrosis through stimulation of extracellular matrix production. Hepatocytes regulate key liver functions including lipid and xenobiotic metabolism, whereas stellate cells, typically quiescent, transdifferentiate during hepatic injury, leading to excessive extracellular matrix (ECM) deposition and fibrosis. Thus, incorporating both cell types is essential for modeling fibrosis.

Cellular adenosine triphosphate levels were unchanged between the spheroids incubated with fatty acid (FA)+TGFB1 and regular medium, indicating that FA and TGFB1 incubation did not affect viability (Figure 1*A*). To measure cell proportions in spheroids, albumin, a hepatocyte marker, and vimentin, a stellate cell marker, were used for immunofluorescence. Quantification showed that the PHHs:PHSCs at the end point was maintained after seeding (Figure 1*B*).

**Figure 1:**
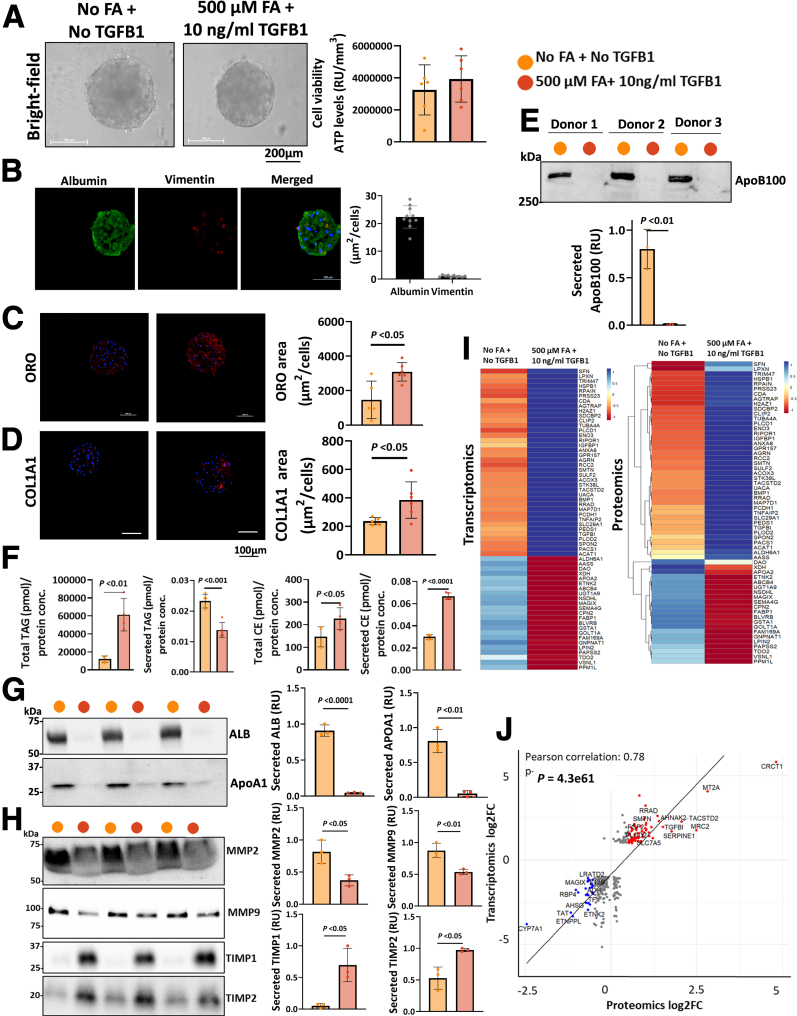
**Fatty acid and TGFB1 incubation in di-lineage primary human hepatic spheroids capture MASLD features.** PHHs and PHSCs were cultured at the ratio of 24:1 in 96-well U-bottom ultra-low attachment plates for 10 days. Twenty-four hours post seeding, spheroids were cultured with either regular media (No FA + No TGFB1) or media supplemented with high fatty acid (500 μM final concentration of oleic and palmitic acid at 2:1 ratio) and 10 ng/mL TGFB1 (FA+TGFB1). (*A*) Bright-field images were captured at day 10, and cell viability was measured using Cell-Titer-Glo (n = 4 for each donor). Data shown as mean ± SD of ATP measurement from 6 donors, normalized to the spheroid volume. *P* value calculated by Mann–Whitney nonparametric test. (*B*) ALB (hepatocyte marker; *green channel*) and vimentin (stellate cell marker; *red channel*) were detected using immunofluorescence. Objective 40×; DAPI: *blue*; ALB and vimentin area were quantified using ImageJ and shown as relative area compared with average vimentin quantification. (*C*) Intracellular neutral lipid content was visualized by ORO staining in di-lineage spheroids; (*D*) COL1A1 was detected by immunofluorescence. Spheroids were fixed in 10% paraformaldehyde for 2 hours and kept with 20% sucrose overnight and then cryo-sectioned into 8-μm sections. Objective: 20×, DAPI, *blue*; ORO, *red* (Texas red); COL1A1, *red* (Alexa594). Scale bar, 100 μm. ORO and COL1A1 images were quantified using ImageJ and normalized to the number of DAPI-stained nuclei. Data are shown as mean ± SD of an average of 10 images from 6 different donors. The *P* values for image quantification were calculated by Mann–Whitney nonparametric test. (*E*) Secreted ApoB100 levels measured by immunoblotting from the cell culture media. (*F*) TAG and CE were quantified using mass spectrometry in the spheroids to get the intracellular total TAG and CE, and 50-μL media to get secreted TAG and CE. TAG and CE are shown in pmol and normalized by the total cellular protein content measured using 280 nm absorbance based nanodrop readings. (*G*) Secreted ALB and secreted ApoA1 in the spheroid culture media were measured by immunoblotting. (*H*) Secreted MMP2, MMP9, TIMP1, and TIMP2 protein levels were measured by immunoblotting in the cell media. All the immunoblots are normalized using total protein as measured using red ponceau. Data is shown as mean ± SD and tested for statistical significance using unpaired Student’s *t* test. Heatmap of the top genes plotted as median log2FC for di-lineage spheroids incubated with or without FA+TGFB1 from (*I*) transcriptomics and proteomics for only those that were differentially expressed in both transcriptomic and proteomic data. The data are clustered based on the protein expression, and the same order was kept for gene expression. (*J*) Correlation of the gene and protein expression (log2FC for the di-lineage spheroids incubated with or without FA + TGFB1). *Red color* represents upregulated genes/proteins in FA + TGFB1 incubated spheroids and *blue color* represents downregulated genes/proteins in FA + TGFB1 incubated spheroids. ALB, albumin; APOA1, apolipoprotein A1; ATP, adenosine triphosphate; CE, cholesteryl ester; COL1A1, collagen type-I alpha-1; DAPI, 4′,6-diamidino-2-phenylindole; FC, fold change; MMP2, matrix metallopeptidase 2; MMP9, matrix metallopeptidase 9; ORO, Oil-Red-O staining; PHH, primary human hepatocyte; PHSC, primary human hepatic stellate cell; RU, relative unit; SD, standard deviation; TAG, triacylglycerol; TIMP1, tissue inhibitor of metalloproteinase 1; TIMP2, tissue inhibitor of metalloproteinase 2.

To account for the human genetic variability, we utilized PHHs and PHSCs from 6 different donors each. Spheroids incubated with FA+TGFB1 resulted in: (1) higher intracellular neutral lipid content, as measured by Oil-Red-O staining (Figure 1*C*); (2) higher levels of collagen type-I alpha-1 (COL1A1) as measured by immunostaining (Figure 1*D*); (3) lower levels of secreted ApoB100 as measured by immunoblotting in the spheroid cultured media (Figure 1*E*; Supplementary Figure 4*A*); (4) higher intracellular triacylglycerol and cholesteryl ester levels, whereas in the culture medium, triacylglycerol levels were lower, and cholesteryl ester levels were higher (Figure 1*F*); (5) lower levels of secreted albumin and apolipoprotein A1 levels (Figure 1*G*; Supplementary Figure 4*C–D*). Secreted matrix metallopeptidase 2 and matrix metallopeptidase 9 were lower, and tissue inhibitor of metalloproteinase 1 and tissue inhibitor of metalloproteinase 2 were higher in the FA+TGFB1 incubated spheroids compared with regular media. These findings indicate a shift towards a profibrotic secretory environment (Figure 1*H*; Supplementary Figure 4*E–H*).

To elucidate molecular changes induced by incubation with FA+TGFB1, we performed RNA sequencing and liquid chromatography-tandem-tandem mass spectrometry proteomic analyses. In total, 10,228 genes were detected by RNA sequencing analysis, whereas 5817 proteins were identified by liquid chromatography-tandem-tandem mass spectrometry proteomics. The overall gene and protein expression differences were visualized using principal component analysis (Supplementary Figure 1*A*). Among these, 3334 genes were common to both transcriptomic and proteomic datasets (Supplementary Figure 1*B*).

Pathway enrichment revealed an upregulation in signaling by TGFB family members and ECM organization, whereas metabolic pathways, specifically biological oxidation and cholesterol metabolism, were downregulated (Supplementary Figure 1*C–D*).

The top differentially expressed genes and proteins, clustered based on protein expression, are shown in the heatmap (Figure 1*I*). Notably, fibrosis-associated markers like TGFBI and procollagen-lysine,2-oxoglutarate 5-dioxygenase 2 were higher at both transcript and protein levels, consistently with their roles in disease progression. Apoliporotein A2, ethanolamine kinase 2, fatty acid binding protein 1, and lipin-2 were lower, suggesting a dysregulation of lipid metabolic pathways in MASLD. Together, these findings underpin the complex interplay between fibrotic remodeling and metabolic disruption in the molecular pathology of MASLD (Figure 1*I*).

The integration of transcriptomic and proteomic data showed a strong correlation between gene expression and protein levels (Pearson correlation coefficient 0.78, *P* <4.3e^−61^). This integrated data highlights several genes of high importance, providing a more nuanced understanding of the molecular mechanisms underlying MASLD (Figure 1*J*). Additionally, the molecular signature obtained provides a more detailed understanding of the underlying metabolic and ECM alterations in FA+TGFB1–incubated spheroids (Supplementary Figure 2).

To validate these findings, we compared our results with the GepLiver database,^7^ which categorizes human liver gene expression by METAVIR fibrosis scores (F0–F4).^7^ The molecular signatures observed in our spheroids showed strong correlation with human disease progression (Figure 2*A*). Specifically, markers such as COL1A1 and actin alpha 2, smooth muscle increased across fibrosis stages, mirroring induction seen in our model (Supplementary Figure 3).

**Figure 2:**
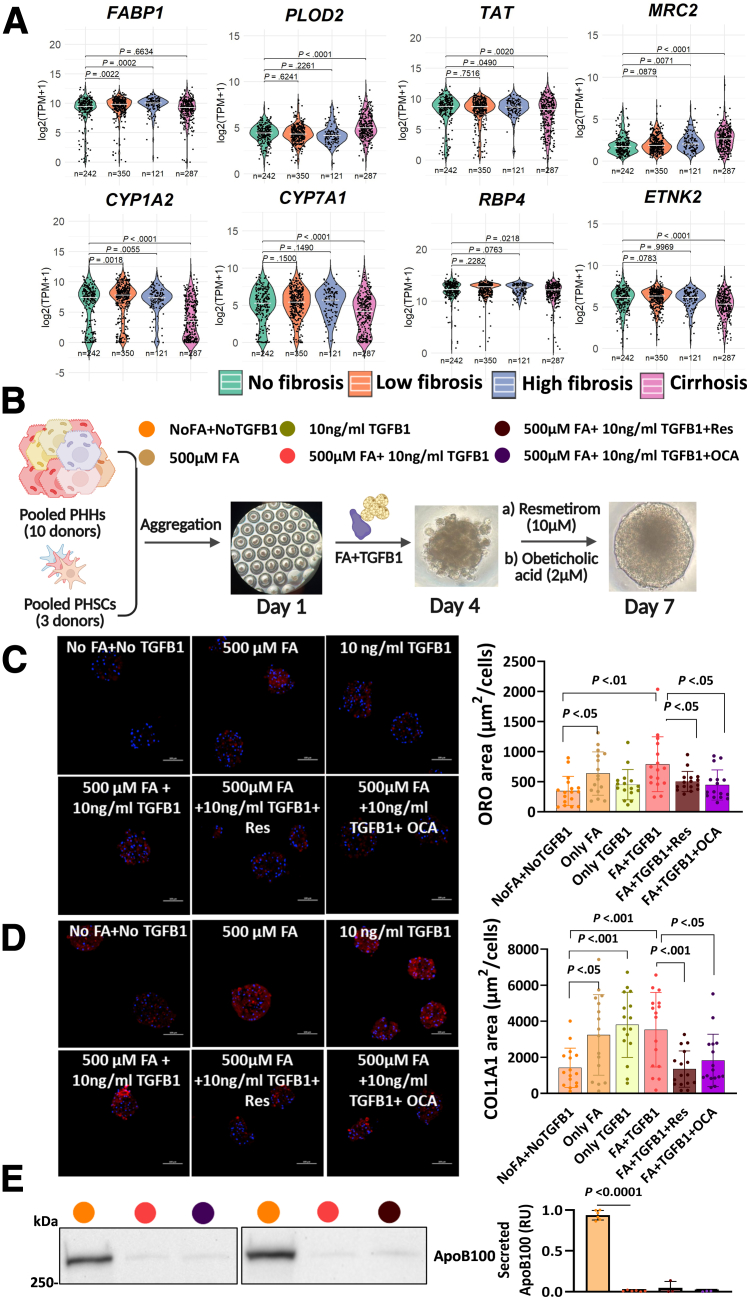
**Comparative omics and phenotypic analysis of MASH spheroids following treatment with resmetirom or obeticholic acid.** (*A*) A comparison between individuals without fibrosis (none) and those with different stages of fibrosis (low, high, and cirrhosis) in the GepLiver database for different genes as reported at the top of each panel. The number of individuals for each group is reported in the plot. *P* values were calculated using unpaired Student’s *t* test. (*B*) Schematic representation of the experiment timeline. Briefly, pooled PHHs from 10 donors and pooled PHSCs from 3 donors were co-cultured at the ratio of 24:1 in 96-well round-bottom Elplasia plates (Corning) for 7 days. Twenty-four hours post seeding, they were cultured with either regular medium or with medium supplemented with high FA (500 μM final concentration of oleic and palmitic acid at 2:1 ratio) and/or 10 ng/ml TGFB1. Then, on day 4 spheroids were treated with resmetirom (10 μM) or obeticholic acid (2 μM), for 3 more days. Then, di-lineage spheroids were fixed in 10% paraformaldehyde for 2 hours, kept with 20% sucrose overnight, and cryosectioned into 8-μm sections. This was followed by (*C*) ORO and (*D*) COL1A1 immunofluorescence. Objective: 20×, DAPI, *blue*; ORO, *red* (Texas red); COL1A1, *red* (Alexa594). Scale bar, 100 μm. ORO and COL1A1 images were quantified using ImageJ and normalized to the number of DAPI-stained nuclei. Data is shown as mean ± SD. The *P* values for image quantification were calculated by Mann–Whitney nonparametric test. (*E*) ApoB100 protein level was measured from the cell culture supernatant, using immunoblotting. Data is shown as mean ± SD and tested for statistical significance using unpaired Student’s *t* test. COL1A1, collagen type-I alpha-1; DAPI, 4′,6-diamidino-2-phenylindole; FA, fatty acid; MASH, metabolic dysfunction-associated steatohepatitis; ORO, Oil-Red-O staining; PHH, primary human hepatocyte; PHSC, primary human hepatic stellate cell; SD, standard deviation; TFGB1, transforming growth factor β 1.

Although the high dimensionality of the proteomics data relative to the sample size limits statistical power for stringent false discovery rate correction, this approach provides a robust discovery framework. A paired Welch’s *t* test was used to account for interindividual variability across 6 donors, with each subject serving as their own reference, reducing noise and improving sensitivity. In addition, multinotch MS3 tandem mass tag labeling, well-suited for small cohorts, minimizes technical variability through multiplexing, and enhances quantitative precision and detection of subtle differences was used. Although stringent false discovery rate correction in high-dimensional datasets with limited sample sizes can mask biologically relevant changes, we addressed this by validating key proteomic hits using orthogonal methods, including transcriptomics, immunofluorescence, and Western blotting. This integrated approach supports the di-lineage model as a valuable platform for studying MASLD and potentially developing antifibrotic therapies.

To assess pharmacologic responsiveness, di-lineage spheroids were generated using PHHs from 10 human donors and pooled PHSCs from 3 donors. Following incubation with FA+TGFB1 for 72 hours, resmetirom (a thyroid hormone receptor β-selective agonist)^8^ or obeticholic acid (a Farnesoid X receptor agonist)^9^ were introduced and incubated for 72 hours (Figure 2*B*). Under these conditions, treatment with either resmetirom or obeticholic acid lowered intracellular neutral lipid accumulation and COL1A1 levels, consistent with human trials; however, ApoB100 secretion was not restored (Figure 2*C–E*; Supplementary Figure 4*B*).

One major limitation of our study is the use of only hepatocytes and hepatic stellate cells, although MASLD involves complex interactions among liver–resident cell populations, including Kupffer cells and liver sinusoidal endothelial cells. Despite this limitation, our model provides a simplified yet robust discovery tool to study core mechanisms underlying MASLD progression.

## Supplementary Material

Supplementary Information

Extended PDF
