# Supplementary material for: Di-Lineage Hepatic Spheroids From Human Donors Capture Steatotic Liver Disease
Source: Cell Mol Gastroenterol Hepatol. 2026 May 8;20(9):101804. doi: 10.1016/j.jcmgh.2026.101804 (PMC13334810; doi:10.1016/j.jcmgh.2026.101804)
Supplement: Supplementary Information [file mmc1.pdf]

Supplementary information:

Methods:

Primary cell culture

Cryopreserved primary human hepatocytes (PHHs) and primary human stellate cells (PHSCs) were purchased from BioIVT or BeCytes, with the characteristics of all primary cells detailed in Supplementary Table1.

Cells were thawed at 37°C and resuspended in 5 ml pre-warmed spheroids plating media (BioIVT). Next, cells were counted using Trypan blue exclusion method on Countess-3 automated cell counter.

For di-lineage spheroid formation, 5000 viable cells were seeded in a 24:1 ratio (PHHs:PHSCs) into 96-well round-bottom ultra-low attachment plates (Corning), using 100 µl of spheroid plating media. Plates were centrifuged at 250g for 3 minutes to facilitate self-aggregation at the bottom of the wells. After 48 hours, an additional 100 µl of spheroid maintenance media (BioIVT) with or without fatty acid supplementation (FA, oleic and palmitic acid in a 2:1 ratio, final concentration 500 µM) and TGFB1 (final concentration of 10 ng/ml) was added to each well. Every 48h, 100 µL fresh spheroid maintenance media was replenished with respective media conditions until day 10. On 10th day, di-lineage spheroids were collected for the subsequent experiments. Due to differences in donor, some spheroids take slightly longer to have compactness, thus the experiment was continued until day 10.

For validation experiment, cryopreserved PHHs from 10 different human donors and PHSCs from 3 donors were seeded in a ratio of 24:1 (PHHs:PHSCs) into each microcavity of 96-well round-bottom Elplasia plates (Corning). A total of 1000 viable cells per microcavity (79,000

cells per well) were seeded in 100 µl spheroid plating media (BioIVT) and gently agitated for 15 minutes, followed by centrifugation at 250g for 3 minutes. After 24 hours, 100 µl of spheroid maintenance media (BioIVT) with or without FA and TGFB1 with above mentioned concentration was added. On day 4, spheroids were additionally incubated with resmetirom (10 µM, MedChemExpress, #HY-12216) or obeticholic acid (2µM, MedChemExpress, #HY-12222), in combination with 500 µM FA+ 10 ng/ml TGFB1. Spheroids were collected on day 7 for analysis.

Cell viability

Total cellular ATP was measured using Cell-Titer-Glo cell viability assay (Promega), as suggested by manufacturer's instructions with slight modifications. Three images per condition were captured using an Axio Vert.A1 inverted microscope (Carl Zeiss AG) to measure the diameters of the spheroids. Cellular ATP was then normalized to the spheroids volume, and the donor averages were used for analysis.

Immunoblotting

The cultured media were mixed with 4x loading buffer and denatured at 95°C for 5 minutes. Immunoblotting for ApoB100 was performed as previously described [1].

For others, primary antibodies used were: mouse anti-ALB (Sigma-Aldrich, A6684, 1:2500), mouse anti-APOA1 (AbD Serotec, 0650 – 0050, 1:1000), MMP2 (Abcam UK, Cat #ab92536, dilution 1:2000), MMP9 (Abcam UK, Cat #ab760003, dilution 1:2000) TIMP1 (Abcam UK, Cat #ab109125, dilution 1:2000), TIMP2 (Abcam UK, Cat #ab180630, dilution 1:500). The membrane was then probed with respective HRP-conjugated secondary antibody (Abcam, Cat #NA931V, dilution 1:2000) for 1h. Blots were developed using ECL high sensitivity substrate

(Immobilon Western Chemiluminescent HRP Substrate, Merck Millipore), visualized using the ChemiDoc Imaging system and quantified using BioRad ImageLab software.

Immunofluorescence

ORO and COL1A1 staining were performed as previously described [2].

For characterization of hepatocytes and hepatic stellate cells, spheroids were incubated with albumin (Sigma-Aldrich, A6684, 1:250) and vimentin (Cell Signaling, D21H3, 1:200) overnight at 4°C followed by probing with anti-mouse secondary antibody conjugated with AlexaFluor488 (Invitrogen, A11001, 1:2000) and anti-rabbit secondary antibody conjugated with AlexaFluor594 (Invitrogen, A21207, 1:2000) for 1h at room temperature. Nuclei were stained with DAPI (Sigma-Aldrich, dilution 1:8000 in PBS) for 5 min. The slides were mounted using fluorescence mounting medium (Dako).

Images were captured using a Nikon microscope with software NIS-Element 5.30.04 (Bergman Labora, Gothenburg, Sweden) at 20x magnification. The stained area was normalized to the number of DAPI-stained nuclei.

Relative Quantitative Proteomics

Proteins were extracted in lysis buffer (2% SDS, 100 mM TEAB) using ultrasonication (ML230, Covaris), and concentrations were determined by BCA assay (Thermo Scientific). Samples (60 µg) were processed using a modified SP3 protocol, including reduction (10 mM DTT, 56°C, 30 min), alkylation (20 mM iodoacetamide, RT, 30 min), and protein capture on Sera-Mag SpeedBeads (Carboxylate-Modified, Cytiva) followed by ethanol precipitation and washing. Proteins were digested with Trypsin/Lys-C [1:25, Promega] for two hours and trypsin [1:50, Promega] overnight at 37°C. Peptides were collected and quantified using Pierce Quantitative

Fluorometric Peptide Assay (Thermo Scientific). Peptide samples (15 µg) including a representative reference pool, were labelled using TMTpro 18-plex reagents (Thermo Fisher Scientific). The labelled samples were pooled into two TMT-set, purified using HiPPR Detergent Removal Resin and Pierce peptide desalting spin columns (both Thermo Scientific) and fractionated by basic reversed-phase chromatography on a XBridge BEH C18 column (3.5 µm, 2.1x250 mm, Waters). Peptides were separated using a stepped gradient from 3% to 67% acetonitrile (ACN) in 25 mM ammonia over 90 min at a flow rate of 200 µL/min, combined into 36 fractions, dried, and reconstituted in 3% ACN and 0.1% trifluoroacetic.

Fractions were analyzed on an Orbitrap Lumos Tribrid mass spectrometer equipped with a FAIMS Pro ion mobility interface and coupled to an Easy-nLC1200 system (all Thermo Fisher Scientific). Peptides were trapped on an Acclaim PepMap 100 C18 trap column (100 µm × 2 cm, 5 µm) and separated on an in-house packed analytical column (35 cm × 75 µm, 3 µm, Reprosil-Pur C18) using a stepped gradient from 5% to 80% ACN in 0.2% formic acid over 85 min at 300 nL/min. FAIMS Pro alternated between compensation voltages of −50 and −70, with identical data-dependent acquisition settings.

Full MS spectra were acquired at 120,000 resolutions over an m/z range of 375–1500. The most abundant precursors (charge 2–7) were selected within a 1.5 s cycle time using a 0.7 m/z isolation window and fragmented by CID (35%), with MS2 spectra acquired in the ion trap (rapid scan rate) and a dynamic exclusion of 60 s. The ten most intense MS2 fragment ions were selected for multi-notch MS3 analysis using HCD (55%), and MS3 spectra were recorded in the Orbitrap at 50,000 resolutions over an m/z range of 100–500.

Raw files were processed using Proteome Discoverer (v3.0, Thermo Fisher Scientific) and searched against the human SwissProt database (20,597 entries, February 2024) using

Sequest. Search parameters included a precursor tolerance of 5 ppm, fragment ion tolerance of 0.6 Da, and up to one missed cleavage. Methionine oxidation was set as a variable modification, while carbamidomethylation and TMTpro labeling of lysine residues and peptide N-termini were set as fixed modifications. PSM validation was performed using Percolator with a 1% FDR threshold. TMT reporter ions were quantified from MS3 HCD spectra with a 3 mmu tolerance and normalized to total peptide abundance. An SPS threshold of 65% and a Sequest HT score cutoff of 2 were applied. Only unique peptides were used for quantification, and proteins were required to meet a 1% FDR.

Transcriptomics

Biomarker Technologies (BMKGENE) performed RNA sequencing. Raw data (raw reads) of fastq format were first processed through in-house perl scripts. In this step, clean data (clean reads) were obtained by removing reads containing adapter or ploy-N, and low-quality reads from raw data. At the same time, Q20, Q30, GC-content and sequence duplication level of the clean data were calculated. All the downstream analyses were based on clean data with high quality.

String Tie (1.3.1) was used to calculate FPKMs (fragments per kilo-base of exon per million fragments mapped) of coding genes in each sample. Gene FPKMs were computed by summing the FPKMs of transcripts in each gene group. The resulting P-values were adjusted using Benjamini and Hochberg's approach for controlling the false discovery rate. Genes with an adjusted P <.01 and absolute value of log2(Fold change) >1 found by DESeq were assigned as differentially expressed.

Analysis of triglycerides and cholesteryl esters

The lipid method was adopted from a previous publication [3] with some modifications. Briefly: Extraction of lipids from the Spheroid pellets and from media was performed by an initial solubilization/homogenization step by adding 0.9% NaCl (aq): MeOH: Chloroform in the ratio [1:20:10] to give a total sample volume of 1 mL in each Eppendorf tube (saline was added first). The tubes were ultrasonicated for 10 minutes to facilitate homogenization. After homogenization 100 µl of sample (10%) was used for the liquid-liquid Bligh-Dyer extraction. The initial monophase solvent composition was [8:20:10] mL of 0.9%NaCl(aq):MeOH:Choloroform. The ratio was adjusted to [18:20:20] to induce phase separation, a lower organic phase and upper aqueous phase. The methanol solvent used during extraction contained 20 ng/ml stable isotope labeled triglycerides (TAG) and cholesteryl ester (CE) standard (EquiSPLASH SKU: 330731, Avanti Research). After the phase separation 1 ml of the lower organic layer was recovered into an LC-vial and evaporated to dryness under a stream of nitrogen. The remaining lipid film was solubilized in 500 µl organic solvent of acetonitrile/isopropanol having a ratio of [2:1], this was used as the injection solvent.

The samples were analyzed using Waters ACQUITY Premier liquid chromatography system with Waters HSS T3 2.1*100 mm column (SKU:186003539). Mobile phase A was methanol with 10 mM ammonium format and mobile phase B was isopropanol with 0.2% formic acid. A 19-minute gradient running between 5%-95% of mobile phase B at a flow of 0.3 mL/min was used. Data was acquired using a Waters Xevo TQ Absolute mass spectrometer detector using a multiple reaction monitoring method for the species expected to be most abundant in each class (CE=12 species and TAG=63 species). A low desolvation temperature of 200°C was used to prevent thermal degradation of the CE lipids. Building the acquisition method and peak integration of raw data was done using the software tools LipidCreator[2] and

Skyline (https://skyline.ms/). Quantification was done in the SAS statistical software and was based on the ratio of the analyte to internal standard area and amount of added internal standard added before the extraction. All TAG species were quantified using the TAG[15:0-18:1(d7)-15:0] internal standard and all CE lipids were quantified using the CE[18:1(d7)] internal standard. Amount of lipids per class was determined by summarizing the individual species. The use of a single internal standard for all lipid species in a class is a limitation for accurate absolute quantification. However, the relative amount can be determined with good linearity and precision which is sufficient for comparing samples run with the same method.

Data analysis

For transcriptomic analysis, differential expression analysis was performed using the DESeq R package (v1.10.1). The resulting P-values were adjusted using Benjamini and Hochberg's approach for controlling FDR. Genes with an adjusted P <.01 and log2(Fold change) >1 found by DESeq were assigned as differentially expressed.

For proteomic quantitative analysis, paired t-test was performed to get the P-values. Due to high biological variability and a small sample size, applying false discovery rate (FDR) was overly stringent, and no proteins passed this threshold. Therefore, proteins with P <.05 were considered significant [4].

Genes and proteins involved in different pathways were selected either from published literature or from the list of genes associated with each pathway in the enrichR databases [5-7]. For the integrative analysis of the transcriptomics and proteomics data, all the significant genes (FDR P <.01 and log2FC>1) and proteins (P <.05) were considered for analysis.

Heatmaps were generated based on protein expression data, with corresponding gene expression values arranged in the same order to facilitate interpretation. A scatter plot was used to visualize the integrative analysis, comparing the transcriptomic Vs. proteomic expression changes following incubation with FA+TGFB1. Log2FC between regular media ("No FA + No TGFB1") and "FA + TGFB1" conditions were calculated and plotted.

Liver tissue gene expression data was obtained from the GepLiver database [8], which stratifies samples into four fibrosis categories: no fibrosis, low fibrosis, high fibrosis, and cirrhosis. Statistical significance was assessed using Student's unpaired t-test. Schematic representation figures were created using Biorender.com.

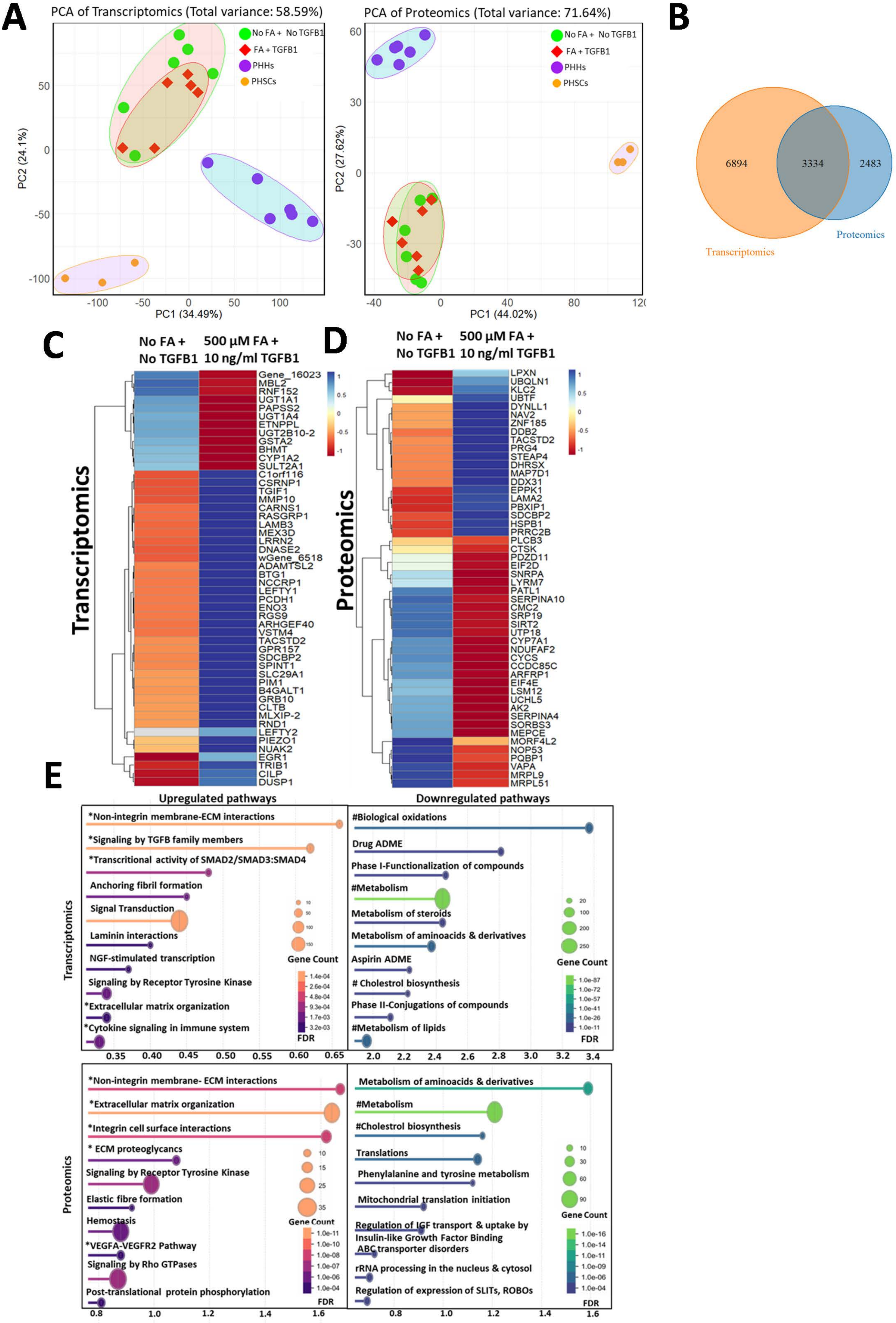
A
PCA of Transcriptomics (Total variance: 58.59%)
No FA + No TGFB1
FA + TGFB1
PHHs
PHSCs
PC2 (24.1%)
PC1 (34.49%)
PCA of Proteomics (Total variance: 71.64%)
PC2 (27.62%)
PC1 (44.02%)
B
6894
3334
2483
Transcriptomics
Proteomics
C
No FA + No TGFB1
500 µM FA + 10 ng/ml TGFB1
Transcriptomics
Gene_16023
MBL2
RNF152
UGT1A1
PAPSS2
UGT1A4
ETNPPL
UGT2B10-2
GSTA2
BHMT
CYP1A2
SULT2A1
C1orf116
CSRNP1
TGIF1
MMP10
CARNS1
RASGRP1
LAMB3
MEX3D
LRRN2
DNASE2
wGene_6518
ADAMTSL2
BTG1
NCCRP1
LEFTY1
PCDH1
ENO3
RGS9
ARHGEF40
VSTM4
TACSTD2
GPR157
SDCBP2
SPINT1
SLC29A1
PIM1
B4GALT1
GRB10
CLTB
MLXIP-2
RND1
LEFTY2
PIEZO1
NUAK2
EGR1
TRIB1
CILP
DUSP1
D
No FA + No TGFB1
500 µM FA + 10 ng/ml TGFB1
Proteomics
LPXN
UBQLN1
KLC2
UBTF
DYNLL1
NAV2
ZNF185
DDB2
TACSTD2
PRG4
STEAP4
DHRSX
MAP7D1
DDX31
EPPK1
LAMA2
PBXIP1
SDCBP2
HSPB1
PRRC2B
PLCB3
CTSK
PDZD11
EIF2D
SNRPA
LYRM7
PATL1
SERPINA10
CMC2
SRP19
SIRT2
UTP18
CYP7A1
NDUFAF2
CYCS
CCDC85C
ARFRP1
EIF4E
LSM12
UCHL5
AK2
SERPINA4
SORBS3
MEPCE
MORF4L2
NOP53
PQBP1
VAPA
MRPL9
MRPL51
E
Upregulated pathways
Downregulated pathways
Transcriptomics
*Non-integrin membrane-ECM interactions
*Signaling by TGFB family members
*Transcritional activity of SMAD2/SMAD3:SMAD4
Anchoring fibril formation
Signal Transduction
Laminin interactions
NGF-stimulated transcription
Signaling by Receptor Tyrosine Kinase
*Extracellular matrix organization
*Cytokine signaling in immune system
Gene Count
FDR
#Biological oxidations
Drug ADME
Phase I-Functionalization of compounds
#Metabolism
Metabolism of steroids
Metabolism of aminoacids & derivatives
Aspirin ADME
Cholestrol biosynthesis
Phase II-Conjugations of compounds
#Metabolism of lipids
Gene Count
FDR
Proteomics
*Non-integrin membrane- ECM interactions
*Extracellular matrix organization
*Integrin cell surface interactions
* ECM proteoglycans
Signaling by Receptor Tyrosine Kinase
Elastic fibre formation
Hemostasis
*VEGFA-VEGFR2 Pathway
Signaling by Rho GTPases
Post-translational protein phosphorylation
Gene Count
FDR
Metabolism of aminoacids & derivatives
#Metabolism
#Cholestrol biosynthesis
Translations
Phenylalanine and tyrosine metabolism
Mitochondrial translation initiation
Regulation of IGF transport & uptake by Insulin-like Growth Factor Binding
ABC transporter disorders
rRNA processing in the nucleus & cytosol
Regulation of expression of SLITs, ROBOs
Gene Count
FDR

Figure S1: Overall distribution and analysis of proteomic and transcriptomic data. Principal component analysis (PCA) of (A) transcriptomic and proteomic data. All the genes or proteins detected using RNA-seq or LC-MS were used to generate the PCA. Six donors for PHHs, three donors for PHSCs, cultured in 2D, as well as the di-lineage spheroids cultured in regular media or with 500 µM FA+ 10ng/ml TGFB1 were processed for proteomics and transcriptomics. Plots were generated using ggplot2 R package. (B) Venn diagram depicting the common genes and protein detected overall in the dataset. The top 50 differentially expressed (C) genes and (D) proteins identified in spheroids incubated with di-lineage hepatic spheroids with 500 µM FA (OA+PA, 2:1) and TGFB1 (10 ng/ml), compared to regular media (no FA + no TGFB1). Data are expressed as median of log2FC values (log2TPM values for transcriptomics and log2-relative protein abundance for proteomics) of 6 donors with or without FA and TGFB1 incubation. (E) Top 10 upregulated and downregulated pathways as enriched in Reactome database (curated from STRING database[9]) using all the significant genes/proteins (detected by transcriptomics or proteomics) either upregulated or downregulated. * and # denotes pathways associated with extracellular matrix and metabolic pathways, respectively. X-axis represents the signal (scores) of the pathways.

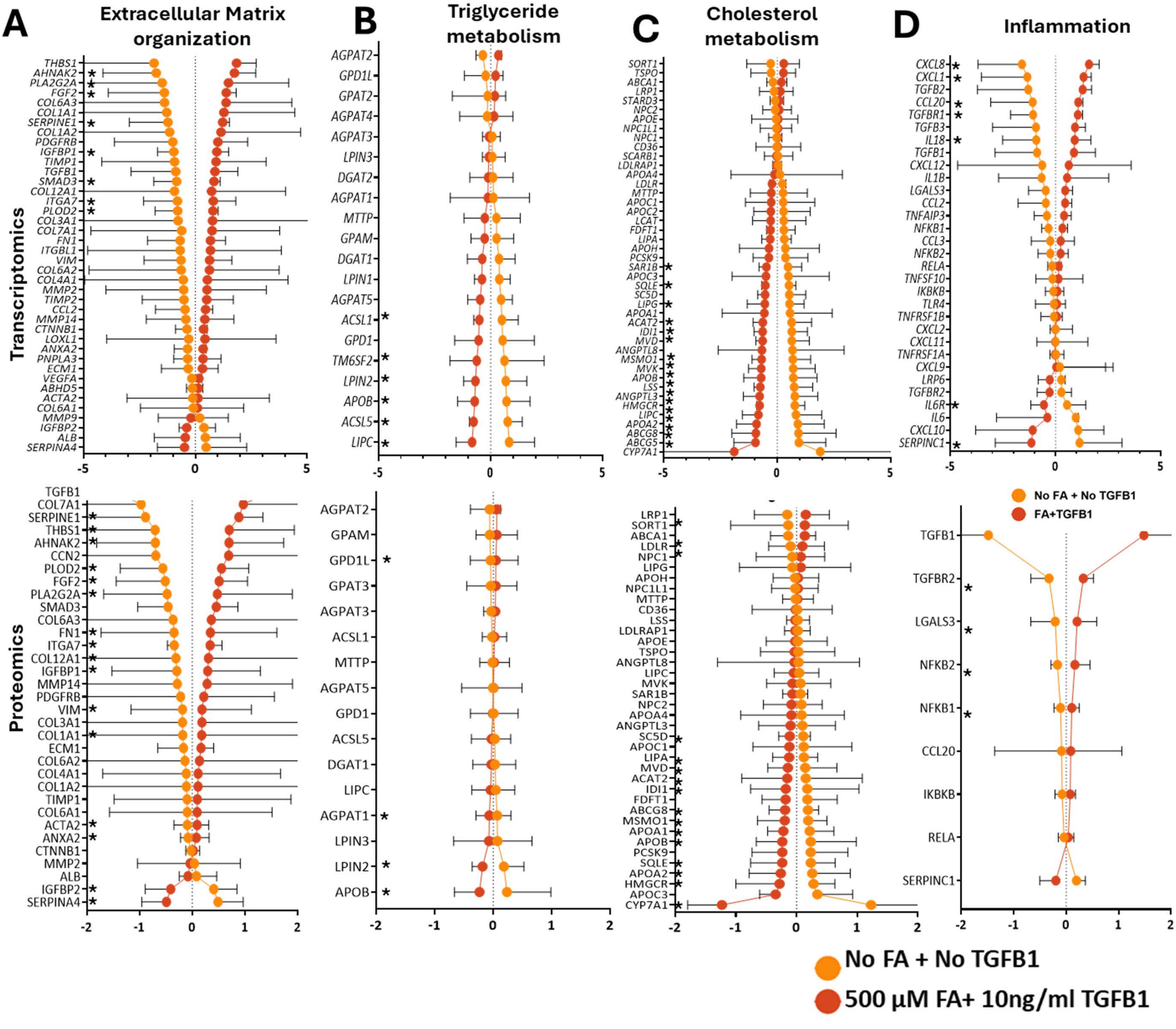

A
Extracellular Matrix organization
B
Triglyceride metabolism
C
Cholesterol metabolism
D
Inflammation
Transcriptomics
Proteomics
THBS1
AHNAK2
PLA2G2A
FGF2
COL6A3
COL1A1
SERPINE1
COL1A2
PDGFRB
IGFBP1
TIMP1
TGFB1
SMAD3
COL12A1
ITGA7
PLOD2
COL3A1
COL7A1
FN1
ITGBL1
VIM
COL6A2
COL4A1
MMP2
TIMP2
CCL2
MMP14
CTNNB1
LOXL1
ANXA2
PNPLA3
ECM1
VEGFA
ABHD5
ACTA2
COL6A1
MMP9
IGFBP2
ALB
SERPINA4
AGPAT2
GPD1L
GPAT2
AGPAT4
AGPAT3
LPIN3
DGAT2
AGPAT1
MTTP
GPAM
DGAT1
LPIN1
AGPAT5
ACSL1
GPD1
TM6SF2
LPIN2
APOB
ACSL5
LIPC
SORT1
TSPO
ABCA1
LRP1
STARD3
NPC2
APOE
NPC1L1
NPC1
CD36
SCARB1
LDLRAP1
APOA4
LDLR
MTTP
APOC1
APOC2
LCAT
FDFT1
LIPA
APOH
PCSK9
SAR1B
APOC3
SQLE
SC5D
LIPG
APOA1
ACAT2
IDI1
MVD
ANGPTL8
MSMO1
MVK
APOB
LSS
ANGPTL3
HMGCR
LIPC
APOA2
ABCG8
ABCG5
CYP7A1
CXCL8
CXCL1
TGFB2
CCL20
TGFBR1
TGFB3
IL18
TGFB1
CXCL12
IL1B
LGALS3
CCL2
TNFAIP3
NFKB1
CCL3
NFKB2
RELA
TNFSF10
IKBKB
TLR4
TNFRSF1B
CXCL2
CXCL11
TNFRSF1A
CXCL9
LRP6
TGFBR2
IL6R
IL6
CXCL10
SERPINC1
-5
0
5
TGFB1
COL7A1
SERPINE1
THBS1
AHNAK2
CCN2
PLOD2
FGF2
PLA2G2A
SMAD3
COL6A3
FN1
ITGA7
COL12A1
IGFBP1
MMP14
PDGFRB
VIM
COL3A1
COL1A1
ECM1
COL6A2
COL4A1
COL1A2
TIMP1
COL6A1
ACTA2
ANXA2
CTNNB1
MMP2
ALB
IGFBP2
SERPINA4
AGPAT2
GPAM
GPD1L
GPAT3
AGPAT3
ACSL1
MTTP
AGPAT5
GPD1
ACSL5
DGAT1
LIPC
AGPAT1
LPIN3
LPIN2
APOB
LRP1
SORT1
ABCA1
LDLR
NPC1
LIPG
APOH
NPC1L1
MTTP
CD36
LSS
LDLRAP1
APOE
TSPO
ANGPTL8
LIPC
MVK
SAR1B
NPC2
APOA4
ANGPTL3
SC5D
APOC1
LIPA
MVD
ACAT2
IDI1
FDFT1
ABCG8
MSMO1
APOA1
APOB
PCSK9
SQLE
APOA2
HMGCR
APOC3
CYP7A1
No FA + No TGFB1
FA+TGFB1
TGFB1
TGFBR2
LGALS3
NFKB2
NFKB1
CCL20
IKBKB
RELA
SERPINC1
-2
-1
0
1
2
No FA + No TGFB1
500 µM FA+ 10ng/ml TGFB1

Figure S2: Genes/proteins related to extracellular matrix, triglyceride, cholesterol metabolism, and inflammation are altered in FA+TGFB1 incubated di-lineage spheroids. Specific genes involved in different pathways related to MASH are plotted from Transcriptomic and proteomic data of di-lineage spheroids incubated with or without FA+TGFB1. (A) extracellular matrix organization, (B) triglyceride metabolism, (C) cholesterol metabolism and (D) inflammation related genes and proteins shown as mean ± SD of log2FC of expression (log2TPM for transcriptomics) and log2(relative protein abundance) for proteomics). P-values calculated using DeSeq2 for transcriptome or paired t-test for proteome data. *P <.05

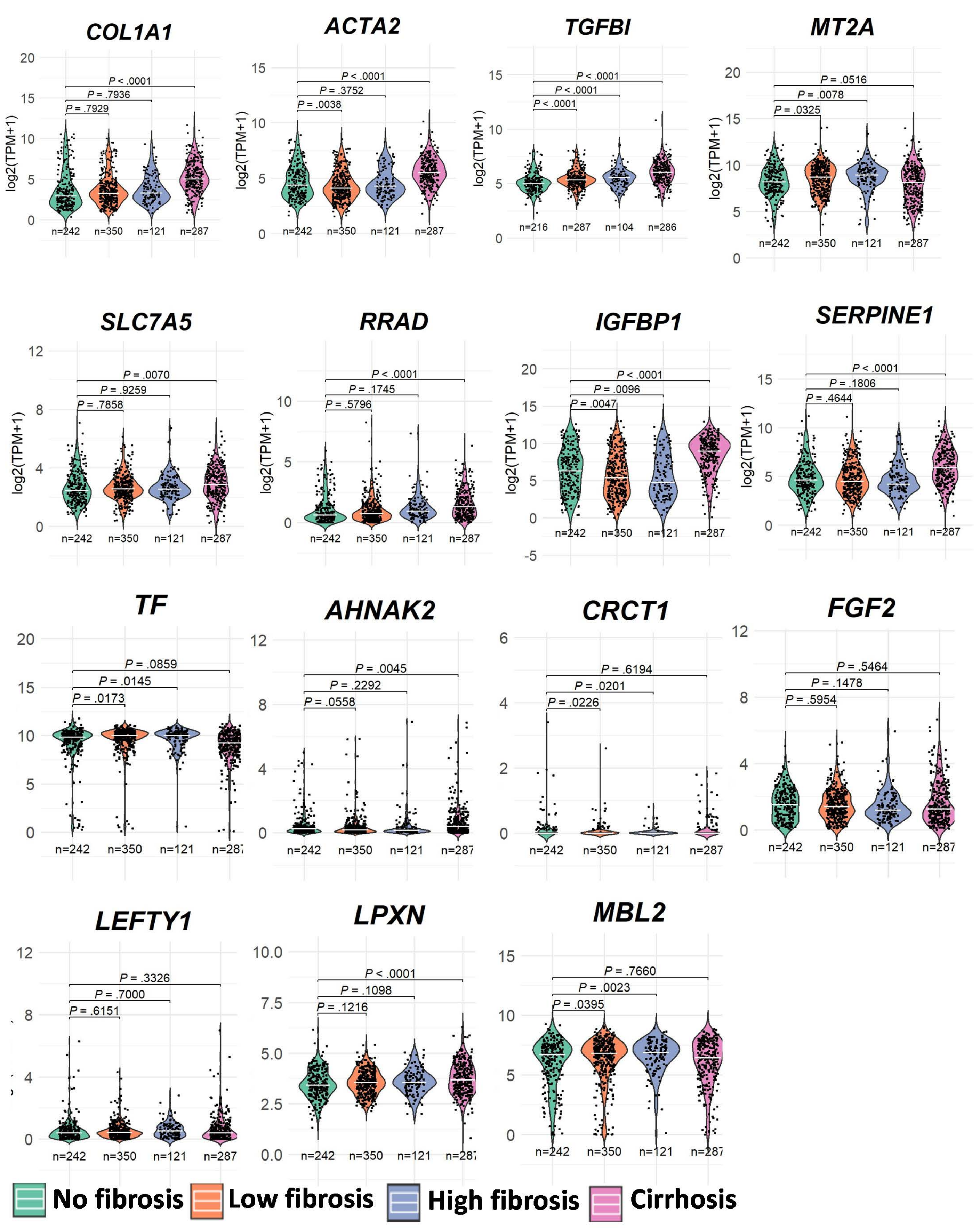
COL1A1
P < .0001
P = .7936
P = .7929
n=242 n=350 n=121 n=287
ACTA2
P < .0001
P = .3752
P = .0038
n=242 n=350 n=121 n=287
TGFBI
P < .0001
P < .0001
P < .0001
n=216 n=287 n=104 n=286
MT2A
P = .0516
P = .0078
P = .0325
n=242 n=350 n=121 n=287
SLC7A5
P = .0070
P = .9259
P = .7858
n=242 n=350 n=121 n=287
RRAD
P < .0001
P = .1745
P = .5796
n=242 n=350 n=121 n=287
IGFBP1
P < .0001
P = .0096
P = .0047
n=242 n=350 n=121 n=287
SERPINE1
P < .0001
P = .1806
P = .4644
n=242 n=350 n=121 n=287
TF
P = .0859
P = .0145
P = .0173
n=242 n=350 n=121 n=287
AHNAK2
P = .0045
P = .2292
P = .0558
n=242 n=350 n=121 n=287
CRCT1
P = .6194
P = .0201
P = .0226
n=242 n=350 n=121 n=287
FGF2
P = .5464
P = .1478
P = .5954
n=242 n=350 n=121 n=287
LEFTY1
P = .3326
P = .7000
P = .6151
n=242 n=350 n=121 n=287
LPXN
P < .0001
P = .1098
P = .1216
n=242 n=350 n=121 n=287
MBL2
P = .7660
P = .0023
P = .0395
n=242 n=350 n=121 n=287
log2(TPM+1)
No fibrosis
Low fibrosis
High fibrosis
Cirrhosis

Figure S3: Comparison of the integrated omics results with data from liver gene expression atlas. A comparison between individuals without fibrosis (No fibrosis) vs. those with different stages of fibrosis (low, high and cirrhosis) in the GepLiver database for different genes as reported at top of each panel. The number of individuals for each group is reported in the plot. P-values were calculated using unpaired Student's t-test.

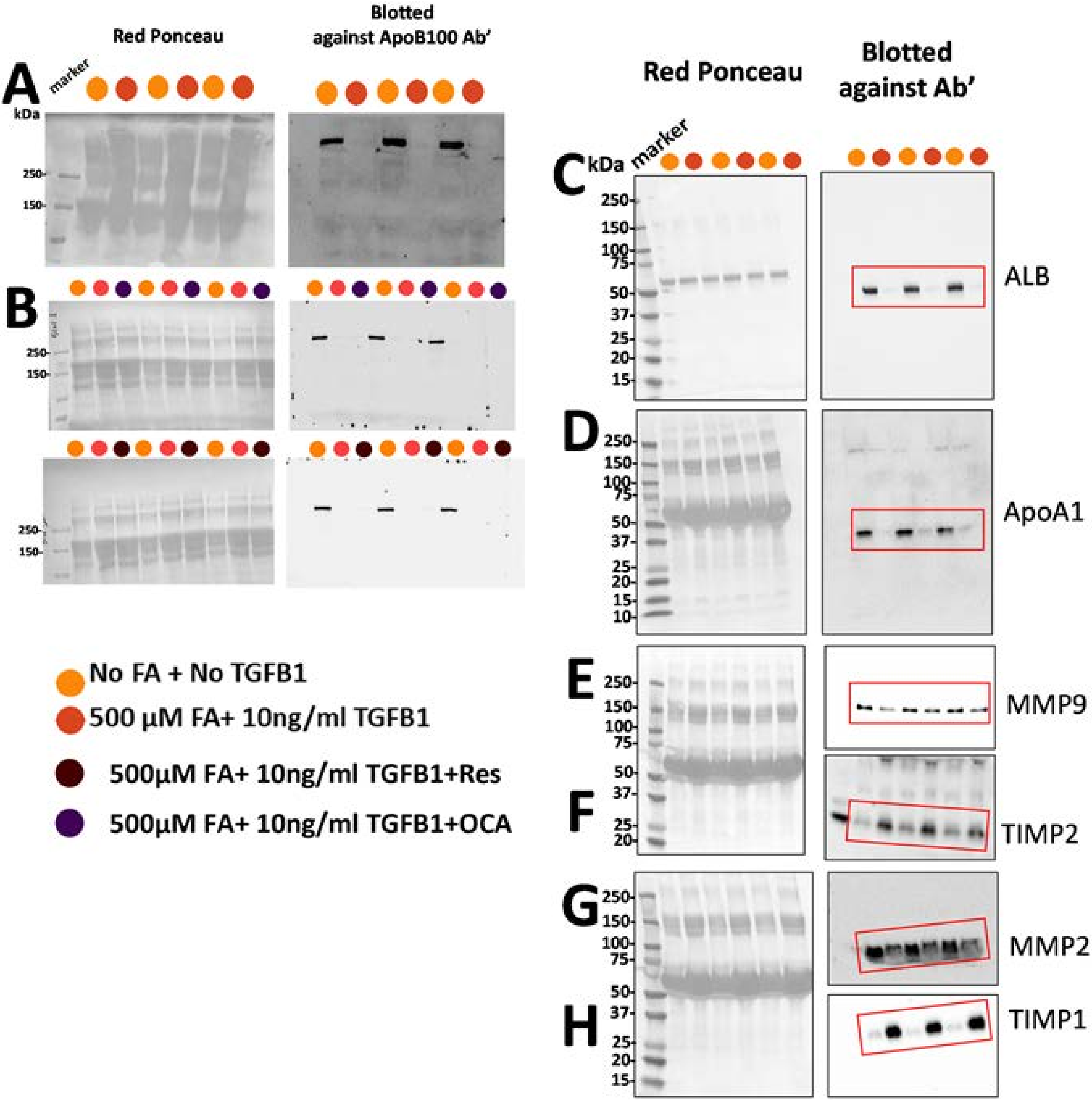

Red Ponceau
Blotted against ApoB100 Ab'
A
marker
kDa
250-
150-
B
250-
150-
250-
150-
No FA + No TGFB1
500 µM FA+ 10ng/ml TGFB1
500µM FA+ 10ng/ml TGFB1+Res
500µM FA+ 10ng/ml TGFB1+OCA
Red Ponceau
Blotted against Ab'
C
kDa
marker
250-
150-
100-
75-
50-
37-
25-
20-
15-
ALB
D
250-
150-
100-
75-
50-
37-
25-
20-
15-
10-
ApoA1
E
250-
150-
100-
75-
MMP9
F
50-
37-
25-
20-
TIMP2
G
250-
150-
100-
75-
MMP2
H
50-
37-
25-
20-
15-
TIMP1

Figure S4: Original Western blot images and corresponding red ponceau images used for protein normalization. Panel A corresponds to figure 1E; panel B corresponds to 2E. Panel C and D corresponds to figure 1G; E-H corresponds to figure 2H. Red marked areas are shown in the main figures.

**Donors**	**Cell types**	**Sex**	**Age**	**Race**	**BMI**	**spheroids combination**
AMC	PHH	F	26	Caucasian	17.6	1
JEL	PHH	F	27	African American	28.2	2
IIN	PHH	F	57	Caucasian	21.3	3
INM	PHH	M	56	Caucasian	22.4	4
OMA	PHH	M	37	Caucasian	24.8	5
BXU	PHH	F	6	Caucasian	22.6	6
XGD	PHSC	F	45	Caucasian	28.1	1
TFE	PHSC	M	50	Caucasian	20.4	2
LVC	PHSC	F	63	Caucasian	39.1	3
BFL	PHSC	F	53	Caucasian	24.5	4
CHM2104-SC-P3-2-Z	PHSC	M	68	Caucasian	25.06	5
CyHuf19006-SC-P2-Z	PHSC	F	65	Caucasian	26.56	6
CyHuf20001-SC-P1-Z	PHSC	F	55	Caucasian	25.2	NA

Supplementary Table 1: Characteristics of primary human hepatocyte and primary hepatic stellate cells used in this study.

References:

1. Sasidharan, K., et al., *IL32 downregulation lowers triglycerides and type I collagen in di-lineage human primary liver organoids.* Cell Rep Med, 2024. **5**(1): p. 101352.
2. Mancina, R.M., et al., *PSD3 downregulation confers protection against fatty liver disease.* Nat Metab, 2022. **4**(1): p. 60-75.
3. Ciociola, E., et al., *Downregulation of the MARC1 p.A165 risk allele reduces hepatocyte lipid content by increasing beta-oxidation.* Clin Mol Hepatol, 2025. **31**(2): p. 445-459.
4. Levin, Y., *The role of statistical power analysis in quantitative proteomics.* Proteomics, 2011. **11**(12): p. 2565-2567.
5. Chen, E.Y., et al., *Enrichr: interactive and collaborative HTML5 gene list enrichment analysis tool.* BMC bioinformatics, 2013. **14**: p. 1-14.
6. Kuleshov, M.V., et al., *Enrichr: a comprehensive gene set enrichment analysis web server 2016 update.* Nucleic acids research, 2016. **44**(W1): p. W90-W97.
7. Xie, Z., et al., *Gene set knowledge discovery with Enrichr.* Current protocols, 2021. **1**(3): p. e90.
8. Li, Z., et al., *GepLiver: an integrative liver expression atlas spanning developmental stages and liver disease phases.* Scientific Data, 2023. **10**(1): p. 376.
9. Szklarczyk, D., et al., *The STRING database in 2023: protein–protein association networks and functional enrichment analyses for any sequenced genome of interest.* Nucleic acids research, 2023. **51**(D1): p. D638-D646.
