# Supplementary material for: Di-Lineage Hepatic Spheroids From Human Donors Capture Steatotic Liver Disease
Source: Cell Mol Gastroenterol Hepatol. 2026 May 8;20(9):101804. doi: 10.1016/j.jcmgh.2026.101804 (PMC13334810; doi:10.1016/j.jcmgh.2026.101804)
Supplement: Extended PDF [file mmc2.pdf]

cmgh
CELLULAR AND MOLECULAR GASTROENTEROLOGY AND HEPATOLOGY

TECHNICAL ADVANCES

Di-Lineage Hepatic Spheroids From Human Donors Capture Steatotic Liver Disease

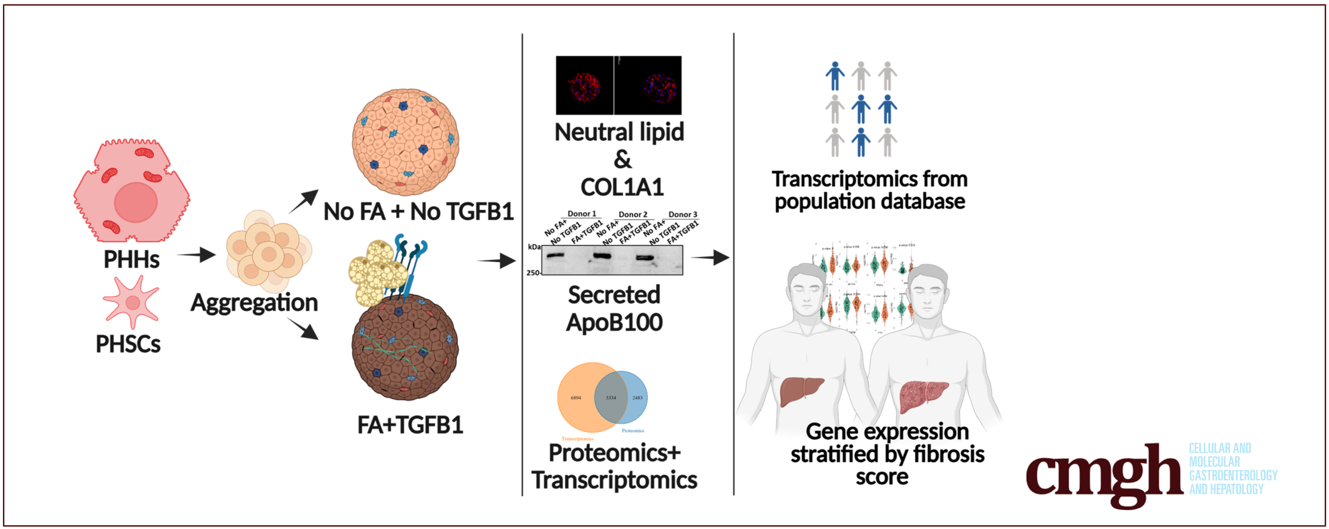

Metabolic dysfunction-associated steatotic liver disease (MASLD) is a prevalent chronic liver condition affecting approximately 32% of the global population.[1] MASLD encompasses a spectrum of liver conditions, ranging from hepatic steatosis to inflammation, steatohepatitis, fibrosis, ultimately leading to cirrhosis and hepatocellular carcinoma.[2,3] Despite its clinical burden, the molecular mechanisms driving the transition from steatosis to metabolic dysfunction-associated steatohepatitis remain poorly understood, hindering the development of reliable biomarkers and effective therapies.

Traditional models, including immortalized cell lines and induced pluripotent stem cell–derived organoids, offer valuable insights but have limitations.[4,5] Spheroids from immortalized cell lines fail to mimic physiological conditions, whereas induced pluripotent stem cell–derived organoids require validation to confirm successful differentiation and appropriate cell type proportions. These limitations underscore the need for advanced 3-dimensional in vitro models bridging between basic research and clinical applications.[6]

To address these shortcomings, we utilized di-lineage primary human spheroids consisting of primary human hepatocytes (PHHs) and primary human hepatic stellate cells (PHSCs) at their physiological ratio (24:1). These spheroids were cultured in regular media or fibrosis-inducing condition using free fatty acids and transforming growth factor β 1 (TGFB1), a known promoter of fibrosis through stimulation of extracellular matrix production. Hepatocytes regulate key liver functions including lipid and xenobiotic metabolism, whereas stellate cells, typically quiescent, transdifferentiate during hepatic injury, leading to excessive extracellular matrix (ECM) deposition and fibrosis. Thus, incorporating both cell types is essential for modeling fibrosis.

Cellular adenosine triphosphate levels were unchanged between the spheroids incubated with fatty acid (FA)+TGFB1 and regular medium, indicating that FA and TGFB1 incubation did not affect viability (Figure 1*A*). To measure cell proportions in spheroids, albumin, a hepatocyte marker, and vimentin, a stellate cell marker, were used for immunofluorescence. Quantification showed that the PHHs:PHSCs at the end point was maintained after seeding (Figure 1*B*).

To account for the human genetic variability, we utilized PHHs and PHSCs from 6 different donors each. Spheroids incubated with FA+TGFB1 resulted in: (1) higher intracellular neutral lipid content, as measured by Oil-Red-O staining (Figure 1*C*); (2) higher levels of collagen type-I alpha-1 (COL1A1) as measured by immunostaining (Figure 1*D*); (3) lower levels of secreted ApoB100 as measured by immunoblotting in the spheroid cultured media (Figure 1*E*; Supplementary Figure 4*A*); (4) higher intracellular triacylglycerol and cholesteryl ester levels, whereas in the culture medium, triacylglycerol levels were lower, and cholesteryl ester levels were higher (Figure 1*F*); (5) lower levels of secreted albumin and apolipoprotein A1 levels (Figure 1*G*; Supplementary Figure 4*C*–*D*). Secreted matrix metallopeptidase 2 and matrix metallopeptidase 9 were lower, and tissue inhibitor of metalloproteinase 1 and tissue inhibitor of metalloproteinase 2 were higher in the FA+TGFB1 incubated spheroids compared with regular media. These findings indicate a shift towards a profibrotic secretory environment (Figure 1*H*; Supplementary Figure 4*E*–*H*).

To elucidate molecular changes induced by incubation with FA+TGFB1, we performed RNA sequencing and

liquid chromatography-tandem-tandem mass spectrometry proteomic analyses. In total, 10,228 genes were detected by RNA sequencing analysis, whereas 5817 proteins were identified by liquid chromatography-tandem-tandem mass spectrometry proteomics. The overall gene and protein expression differences were visualized using principal component analysis (Supplementary Figure 1*A*). Among these, 3334 genes were common to both transcriptomic and proteomic datasets (Supplementary Figure 1*B*).

Pathway enrichment revealed an upregulation in signaling by TGFB family members and ECM organization, whereas metabolic pathways, specifically biological oxidation and cholesterol metabolism, were downregulated (Supplementary Figure 1*C*–*D*).

The top differentially expressed genes and proteins, clustered based on protein expression, are shown in the heatmap (Figure 1*I*). Notably, fibrosis-associated markers like TGFBI and procollagen-lysine,2-oxoglutarate 5-dioxygenase 2 were higher at both transcript and protein levels,

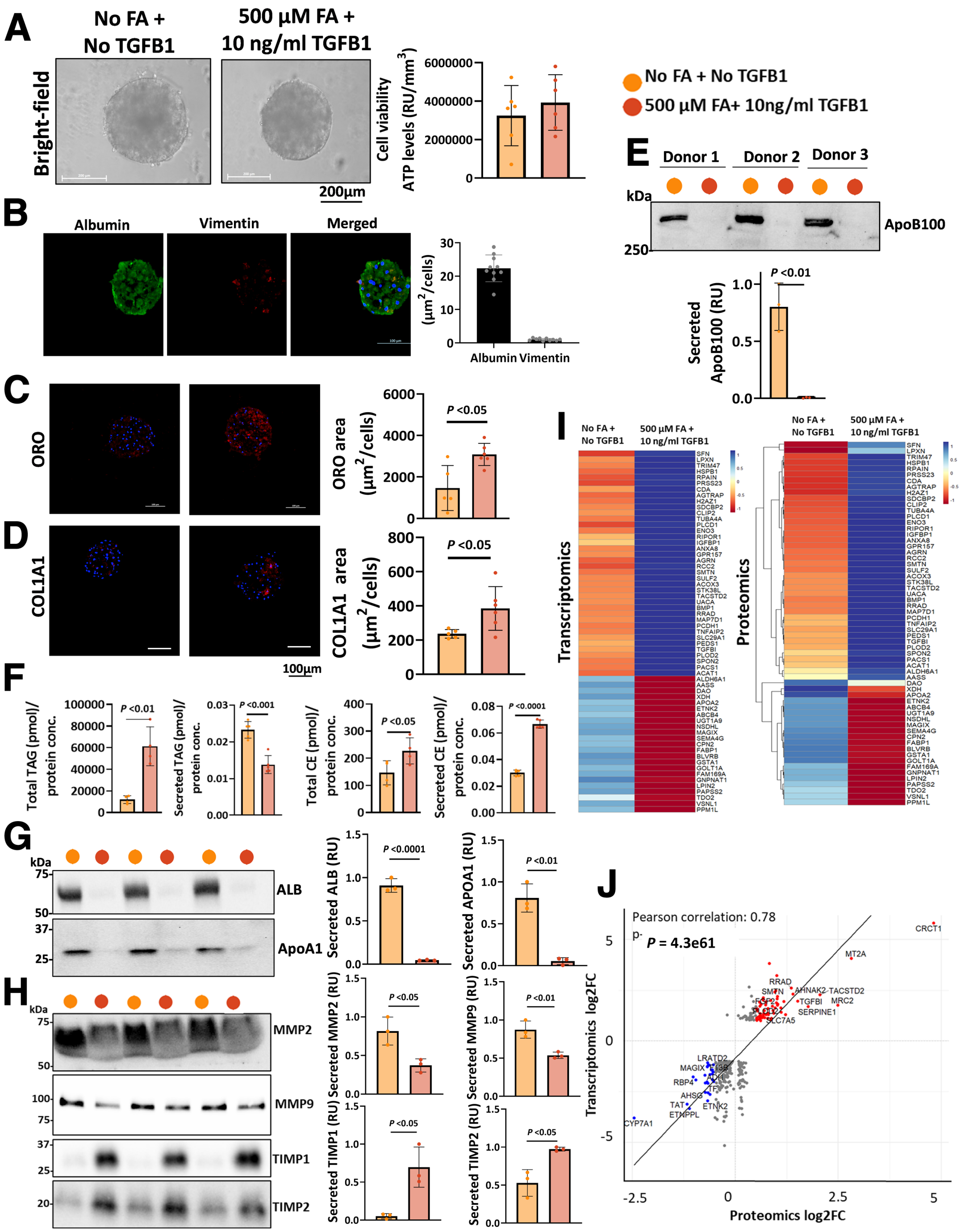

consistently with their roles in disease progression. Apoliporotein A2, ethanolamine kinase 2, fatty acid binding protein 1, and lipin-2 were lower, suggesting a dysregulation of lipid metabolic pathways in MASLD. Together, these findings underpin the complex interplay between fibrotic remodeling and metabolic disruption in the molecular pathology of MASLD (Figure 1*I*).

The integration of transcriptomic and proteomic data showed a strong correlation between gene expression and protein levels (Pearson correlation coefficient 0.78, $P < 4.3e^{-61}$). This integrated data highlights several genes of high importance, providing a more nuanced understanding of the molecular mechanisms underlying MASLD (Figure 1*J*). Additionally, the molecular signature obtained provides a more detailed understanding of the underlying metabolic and ECM alterations in FA+TGFB1–incubated spheroids (Supplementary Figure 2).

To validate these findings, we compared our results with the GepLiver database,[7] which categorizes human liver gene expression by METAVIR fibrosis scores (F0–F4).[7] The molecular signatures observed in our spheroids showed strong correlation with human disease progression (Figure 2*A*). Specifically, markers such as COL1A1 and actin alpha 2, smooth muscle increased across fibrosis stages, mirroring induction seen in our model (Supplementary Figure 3).

Although the high dimensionality of the proteomics data relative to the sample size limits statistical power for stringent false discovery rate correction, this approach provides a robust discovery framework. A paired Welch's *t* test was used to account for interindividual variability across 6 donors, with each subject serving as their own reference, reducing noise and improving sensitivity. In addition, multinotch MS3 tandem mass tag labeling, well-suited for small cohorts, minimizes technical variability through multiplexing, and enhances quantitative precision and detection of subtle differences was used. Although stringent false discovery rate correction in high-dimensional datasets with limited sample sizes can mask biologically relevant changes, we addressed this by validating key proteomic hits using orthogonal methods, including transcriptomics, immunofluorescence, and Western blotting. This integrated approach supports the di-lineage model as a valuable platform for studying MASLD and potentially developing antifibrotic therapies.

To assess pharmacologic responsiveness, di-lineage spheroids were generated using PHHs from 10 human donors and pooled PHSCs from 3 donors. Following incubation with FA+TGFB1 for 72 hours, resmetirom (a thyroid hormone receptor β-selective agonist)[8] or obeticholic acid (a Farnesoid X receptor agonist)[9] were introduced and incubated for 72 hours (Figure 2*B*). Under these conditions, treatment with either resmetirom or obeticholic acid lowered intracellular neutral lipid accumulation and COL1A1 levels, consistent with human trials; however, ApoB100 secretion

Figure 1. (***See previous page***). **Fatty acid and TGFB1 incubation in di-lineage primary human hepatic spheroids capture MASLD features.** PHHs and PHSCs were cultured at the ratio of 24:1 in 96-well U-bottom ultra-low attachment plates for 10 days. Twenty-four hours post seeding, spheroids were cultured with either regular media (No FA + No TGFB1) or media supplemented with high fatty acid (500 μM final concentration of oleic and palmitic acid at 2:1 ratio) and 10 ng/mL TGFB1 (FA+TGFB1). (*A*) Bright-field images were captured at day 10, and cell viability was measured using Cell-Titer-Glo (n = 4 for each donor). Data shown as mean ± SD of ATP measurement from 6 donors, normalized to the spheroid volume. *P* value calculated by Mann–Whitney nonparametric test. (*B*) ALB (hepatocyte marker; *green channel*) and vimentin (stellate cell marker; *red channel*) were detected using immunofluorescence. Objective 40×; DAPI: *blue*; ALB and vimentin area were quantified using ImageJ and shown as relative area compared with average vimentin quantification. (*C*) Intracellular neutral lipid content was visualized by ORO staining in di-lineage spheroids; (*D*) COL1A1 was detected by immunofluorescence. Spheroids were fixed in 10% paraformaldehyde for 2 hours and kept with 20% sucrose overnight and then cryo-sectioned into 8-μm sections. Objective: 20×, DAPI, *blue*; ORO, *red* (Texas red); COL1A1, *red* (Alexa594). Scale bar, 100 μm. ORO and COL1A1 images were quantified using ImageJ and normalized to the number of DAPI-stained nuclei. Data are shown as mean ± SD of an average of 10 images from 6 different donors. The *P* values for image quantification were calculated by Mann–Whitney nonparametric test. (*E*) Secreted ApoB100 levels measured by immunoblotting from the cell culture media. (*F*) TAG and CE were quantified using mass spectrometry in the spheroids to get the intracellular total TAG and CE, and 50-μL media to get secreted TAG and CE. TAG and CE are shown in pmol and normalized by the total cellular protein content measured using 280 nm absorbance based nanodrop readings. (*G*) Secreted ALB and secreted ApoA1 in the spheroid culture media were measured by immunoblotting. (*H*) Secreted MMP2, MMP9, TIMP1, and TIMP2 protein levels were measured by immunoblotting in the cell media. All the immunoblots are normalized using total protein as measured using red ponceau. Data is shown as mean ± SD and tested for statistical significance using unpaired Student's *t* test. Heatmap of the top genes plotted as median log2FC for di-lineage spheroids incubated with or without FA+TGFB1 from (*I*) transcriptomics and proteomics for only those that were differentially expressed in both transcriptomic and proteomic data. The data are clustered based on the protein expression, and the same order was kept for gene expression. (*J*) Correlation of the gene and protein expression (log2FC for the di-lineage spheroids incubated with or without FA + TGFB1). *Red color* represents upregulated genes/proteins in FA + TGFB1 incubated spheroids and *blue color* represents downregulated genes/proteins in FA + TGFB1 incubated spheroids. ALB, albumin; APOA1, apolipoprotein A1; ATP, adenosine triphosphate; CE, cholesteryl ester; COL1A1, collagen type-I alpha-1; DAPI, 4′,6-diamidino-2-phenylindole; FC, fold change; MMP2, matrix metallopeptidase 2; MMP9, matrix metallopeptidase 9; ORO, Oil-Red-O staining; PHH, primary human hepatocyte; PHSC, primary human hepatic stellate cell; RU, relative unit; SD, standard deviation; TAG, triacylglycerol; TIMP1, tissue inhibitor of metalloproteinase 1; TIMP2, tissue inhibitor of metalloproteinase 2.

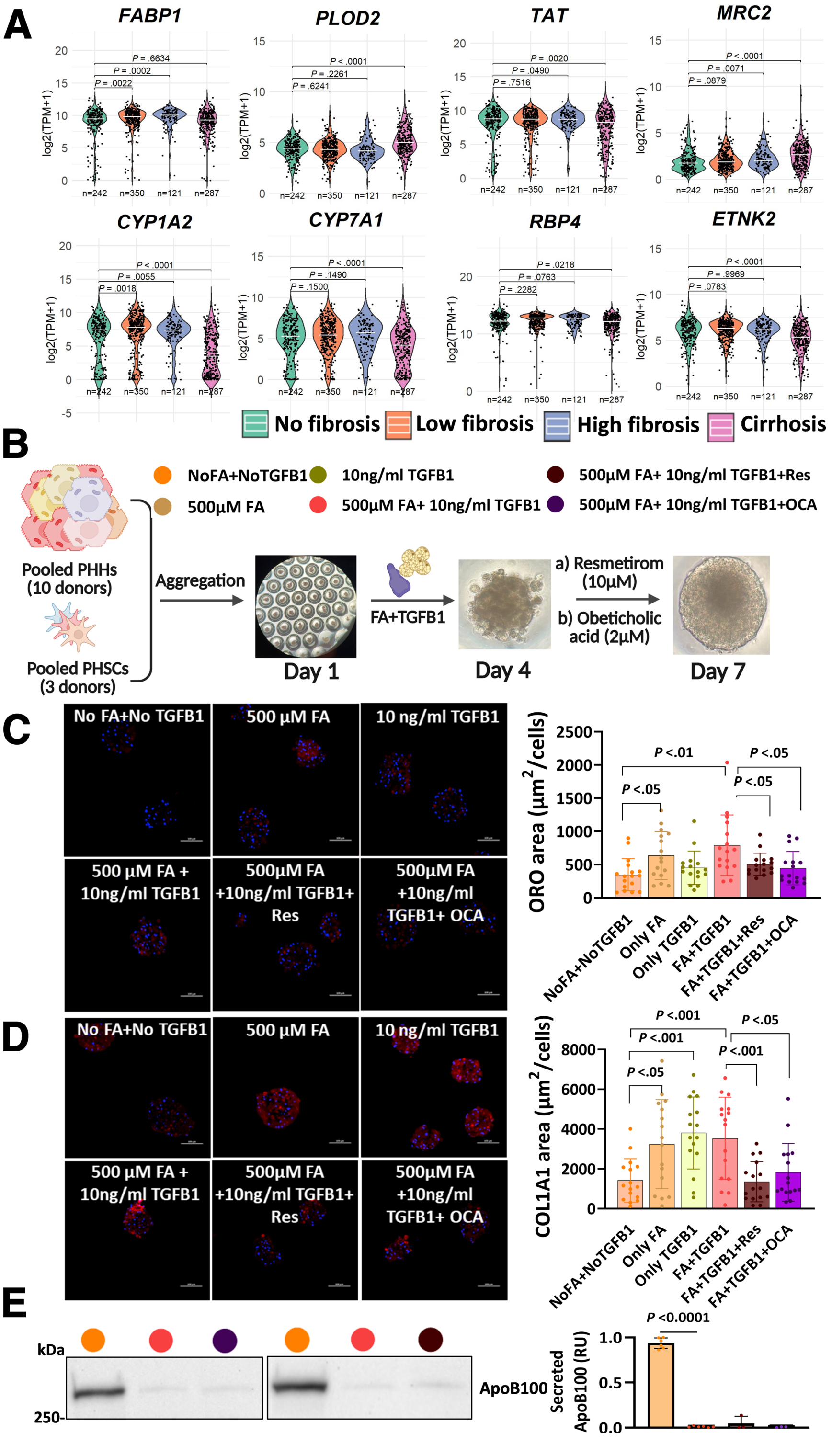

Figure 2. Comparative omics and phenotypic analysis of MASH spheroids following treatment with resmetirom or obeticholic acid. (*A*) A comparison between individuals without fibrosis (none) and those with different stages of fibrosis (low, high, and cirrhosis) in the GepLiver database for different genes as reported at the top of each panel. The number of individuals for each group is reported in the plot. *P* values were calculated using unpaired Student's *t* test. (*B*) Schematic representation of the experiment timeline. Briefly, pooled PHHs from 10 donors and pooled PHSCs from 3 donors were co-cultured at the ratio of 24:1 in 96-well round-bottom Elplasia plates (Corning) for 7 days. Twenty-four hours post seeding, they were cultured with either regular medium or with medium supplemented with high FA (500 μM final concentration of oleic and palmitic acid at 2:1 ratio) and/or 10 ng/ml TGFB1. Then, on day 4 spheroids were treated with resmetirom (10 μM) or obeticholic acid (2 μM), for 3 more days. Then, di-lineage spheroids were fixed in 10% paraformaldehyde for 2 hours, kept with 20% sucrose overnight, and cryosectioned into 8-μm sections. This was followed by (*C*) ORO and (*D*) COL1A1 immunofluorescence. Objective: 20×, DAPI, *blue*; ORO, *red* (Texas red); COL1A1, *red* (Alexa594). Scale bar, 100 μm. ORO and COL1A1 images were quantified using ImageJ and normalized to the number of DAPI-stained nuclei. Data is shown as mean ± SD. The *P* values for image quantification were calculated by Mann–Whitney nonparametric test. (*E*) ApoB100 protein level was measured from the cell culture supernatant, using immunoblotting. Data is shown as mean ± SD and tested for statistical significance using unpaired Student's *t* test. COL1A1, collagen type-I alpha-1; DAPI, 4′,6-diamidino-2-phenylindole; FA, fatty acid; MASH, metabolic dysfunction-associated steatohepatitis; ORO, Oil-Red-O staining; PHH, primary human hepatocyte; PHSC, primary human hepatic stellate cell; SD, standard deviation; TFGB1, transforming growth factor β 1.

was not restored (Figure 2*C*–*E*; Supplementary Figure 4*B*).

One major limitation of our study is the use of only hepatocytes and hepatic stellate cells, although MASLD involves complex interactions among liver–resident cell populations, including Kupffer cells and liver sinusoidal endothelial cells. Despite this limitation, our model provides a simplified yet robust discovery tool to study core mechanisms underlying MASLD progression.

TANMOY DUTTA[*]
Department of Molecular and Clinical Medicine
Institute of Medicine University of Gothenburg
Gothenburg, Sweden

LOHITESH KOVOORU[*]
Department of Molecular and Clinical Medicine
Institute of Medicine University of Gothenburg
Gothenburg, Sweden, *and*
Department of Medicine (H7)
Centre for Reproduction, Metabolism, and Molecular medicine (CeRM)
Karolinska Institute
Huddinge, Sweden

ANNIKA THORSELL
Proteomics Core Facility
BioMS
SciLifeLab
University of Gothenburg
Gothenburg, Sweden

CARMELO PUJIA
SAMANTHA MAUROTTI
Department of Medical and Surgical Sciences
University Magna Graecia
Catanzaro, Italy

KAVITHA SASIDHARAN
Ribocure Pharmaceuticals AB Gothenburg
Gothenburg, Sweden

ROSELLINA M. MANCINA
Department of Molecular and Clinical Medicine
Institute of Medicine University of Gothenburg
Gothenburg, Sweden, *and*
Department of Medicine (H7)
Centre for Reproduction, Metabolism, and Molecular medicine (CeRM)
Karolinska Institute
Huddinge, Sweden, *and*
Department of Life Science, Health, and Health Professions
Link Campus University
Rome, Italy, *and*
Research Unit of Clinical Medicine and Hepatology
Department of Medicine and Surgery
Università Campus Bio-Medico di Roma
Rome, Italy

STEFANO ROMEO
Department of Molecular and Clinical Medicine
Institute of Medicine University of Gothenburg
Gothenburg, Sweden, *and*
Department of Medicine (H7)
Centre for Reproduction, Metabolism, and Molecular medicine (CeRM)
Karolinska Institute
Huddinge, Sweden, *and*
Department of Medical and Surgical Sciences
University Magna Graecia
Catanzaro, Italy, *and*
Department of Cardiology
Sahlgrenska University Hospital
Gothenburg, Sweden, *and*
Department of Endocrinology
Karolinska University Hospital
Huddinge, Sweden

Supplementary Material

Note: To access the supplementary material accompanying this article, visit the full text version at https://doi.org/10.1016/j.jcmgh.2026.101804.

References

1. Teng ML, et al. Clin Mol Hepatol 2022;29(Suppl):S32.
2. Fernando DH, et al. Int J Mol Sci 2019;20:5037.
3. Pelusi S, et al. Liver Int 2019; 39:250–256.
4. Cerneckis J, et al. Signal Transduct Target Ther 2024;9:112.
5. Xu Z, et al. Front Cell Dev Biol 2023;11:1188905.
6. Caddeo A, et al. Atherosclerosis 2024;393:117544.
7. Li Z, et al. Sci Data 2023;10:376.
8. Cusi K. N Engl J Med 2024; 390:559–561.
9. Zhang Y, et al. Pharmacol Res Perspect 2017;5:e00329.

***Authors share co-first authorship.**

© 2026 The Author(s). Published by Elsevier Inc. on behalf of American Gastroenterological Association Institute. This is an open access article under the CC BY license (http://creativecommons.org/licenses/by/4.0/).
2352-345X
https://doi.org/10.1016/j.jcmgh.2026.101804

Correspondence
Address correspondence to: Stefano Romeo, MD, PhD, Department of Medicine, Karolinska Institute and Hospital, Blickagången 16, Huddinge 141 52, Sweden. e-mail: stefano.romeo@ki.se.

Acknowledgments
The authors acknowledge GepLiver for curating liver gene expression data. Transcriptomic analysis was done by BMKgene. Proteomic analysis was performed at the Proteomics Core Facility, Sahlgrenska Academy, Gothenburg University, with financial support from SciLifeLab and BioMS. The SciLifeLab Metabolomics platform, Swedish National Infrastructure for Biological Mass Spectrometry (BioMS), and Chalmers Mass Spectrometry Infrastructure (CMSI) are acknowledged for their support. We also wish to acknowledge Per Larsson at CMSI, who performed the lipid analysis, and Otto Savolainen for his support.

Conflicts of interest
This author discloses the following: In the last 5 years, Stefano Romeo received research grants from Novonordisk and AstraZeneca for basic science research on steatotic liver disease; has been consulting for AstraZeneca, GSK, Celgene Corporation, Ribocure AB, Madrigal, Ultragenyx, Amgen, Sanofi, Wave Life Sciences, Lipigon, Novartis, Profluent, Aina, Echosense, and Chiesi; declares equity from Heptabio; and is inventor on a Patent with title "Method for treating fatty liver disease," on PSD3, U.S. application number 17,480266 filed on 21st September 2021. The remaining authors disclose no conflicts.

Funding
Stefano Romeo was supported by the Region Stockholm (ALF project grant, FoUI-1021801), the Swedish Cancerfonden (22 2270 Pj), the Swedish Research Council (Vetenskapsradet (VR), 2023-02079), the Swedish Heart Lung Foundation (20220334), the Novonordisk Distinguished Investigator Grant - Endocrinology and Metabolism (NNF23OC0082114), the Novonordisk Project grants in Endocrinology and Metabolism (NNF 24OC0091535), and a Novo Nordisk donation to the Karolinska Institutet in connection with the professor appointment.

Data Availability
Transcriptomics and proteomics data for the spheroids are available to download from https://github.com/deccan-barb/Di-Lineage-Hepatic-Spheroids-From-Human-Donors.

Cellular and Molecular Gastroenterology and Hepatology, Volume *20*

Supplemental information

Di-Lineage Hepatic Spheroids From Human Donors Capture Steatotic Liver Disease

Tanmoy Dutta;Lohitesh Kovooru;Annika Thorsell;Carmelo Pujia, Samantha Maurotti;Kavitha Sasidharan;Rosellina M. Mancina;, and Stefano Romeo

Supplementary information:

Methods:

Primary cell culture

Cryopreserved primary human hepatocytes (PHHs) and primary human stellate cells (PHSCs) were purchased from BioIVT or BeCytes, with the characteristics of all primary cells detailed in Supplementary Table1.

Cells were thawed at 37°C and resuspended in 5 ml pre-warmed spheroids plating media (BioIVT). Next, cells were counted using Trypan blue exclusion method on Countess-3 automated cell counter.

For di-lineage spheroid formation, 5000 viable cells were seeded in a 24:1 ratio (PHHs:PHSCs) into 96-well round-bottom ultra-low attachment plates (Corning), using 100 µl of spheroid plating media. Plates were centrifuged at 250g for 3 minutes to facilitate self-aggregation at the bottom of the wells. After 48 hours, an additional 100 µl of spheroid maintenance media (BioIVT) with or without fatty acid supplementation (FA, oleic and palmitic acid in a 2:1 ratio, final concentration 500 µM) and TGFB1 (final concentration of 10 ng/ml) was added to each well. Every 48h, 100 µL fresh spheroid maintenance media was replenished with respective media conditions until day 10. On 10th day, di-lineage spheroids were collected for the subsequent experiments. Due to differences in donor, some spheroids take slightly longer to have compactness, thus the experiment was continued until day 10.

For validation experiment, cryopreserved PHHs from 10 different human donors and PHSCs from 3 donors were seeded in a ratio of 24:1 (PHHs:PHSCs) into each microcavity of 96-well round-bottom Elplasia plates (Corning). A total of 1000 viable cells per microcavity (79,000

cells per well) were seeded in 100 µl spheroid plating media (BioIVT) and gently agitated for 15 minutes, followed by centrifugation at 250g for 3 minutes. After 24 hours, 100 µl of spheroid maintenance media (BioIVT) with or without FA and TGFB1 with above mentioned concentration was added. On day 4, spheroids were additionally incubated with resmetirom (10 µM, MedChemExpress, #HY-12216) or obeticholic acid (2µM, MedChemExpress, #HY-12222), in combination with 500 µM FA+ 10 ng/ml TGFB1. Spheroids were collected on day 7 for analysis.

Cell viability

Total cellular ATP was measured using Cell-Titer-Glo cell viability assay (Promega), as suggested by manufacturer's instructions with slight modifications. Three images per condition were captured using an Axio Vert.A1 inverted microscope (Carl Zeiss AG) to measure the diameters of the spheroids. Cellular ATP was then normalized to the spheroids volume, and the donor averages were used for analysis.

Immunoblotting

The cultured media were mixed with 4x loading buffer and denatured at 95°C for 5 minutes. Immunoblotting for ApoB100 was performed as previously described [1].

For others, primary antibodies used were: mouse anti-ALB (Sigma-Aldrich, A6684, 1:2500), mouse anti-APOA1 (AbD Serotec, 0650 – 0050, 1:1000), MMP2 (Abcam UK, Cat #ab92536, dilution 1:2000), MMP9 (Abcam UK, Cat #ab760003, dilution 1:2000) TIMP1 (Abcam UK, Cat #ab109125, dilution 1:2000), TIMP2 (Abcam UK, Cat #ab180630, dilution 1:500). The membrane was then probed with respective HRP-conjugated secondary antibody (Abcam, Cat #NA931V, dilution 1:2000) for 1h. Blots were developed using ECL high sensitivity substrate

(Immobilon Western Chemiluminescent HRP Substrate, Merck Millipore), visualized using the ChemiDoc Imaging system and quantified using BioRad ImageLab software.

Immunofluorescence

ORO and COL1A1 staining were performed as previously described [2].

For characterization of hepatocytes and hepatic stellate cells, spheroids were incubated with albumin (Sigma-Aldrich, A6684, 1:250) and vimentin (Cell Signaling, D21H3, 1:200) overnight at 4°C followed by probing with anti-mouse secondary antibody conjugated with AlexaFluor488 (Invitrogen, A11001, 1:2000) and anti-rabbit secondary antibody conjugated with AlexaFluor594 (Invitrogen, A21207, 1:2000) for 1h at room temperature. Nuclei were stained with DAPI (Sigma-Aldrich, dilution 1:8000 in PBS) for 5 min. The slides were mounted using fluorescence mounting medium (Dako).

Images were captured using a Nikon microscope with software NIS-Element 5.30.04 (Bergman Labora, Gothenburg, Sweden) at 20x magnification. The stained area was normalized to the number of DAPI-stained nuclei.

Relative Quantitative Proteomics

Proteins were extracted in lysis buffer (2% SDS, 100 mM TEAB) using ultrasonication (ML230, Covaris), and concentrations were determined by BCA assay (Thermo Scientific). Samples (60 µg) were processed using a modified SP3 protocol, including reduction (10 mM DTT, 56°C, 30 min), alkylation (20 mM iodoacetamide, RT, 30 min), and protein capture on Sera-Mag SpeedBeads (Carboxylate-Modified, Cytiva) followed by ethanol precipitation and washing. Proteins were digested with Trypsin/Lys-C [1:25, Promega] for two hours and trypsin [1:50, Promega] overnight at 37°C. Peptides were collected and quantified using Pierce Quantitative

Fluorometric Peptide Assay (Thermo Scientific). Peptide samples (15 µg) including a representative reference pool, were labelled using TMTpro 18-plex reagents (Thermo Fisher Scientific). The labelled samples were pooled into two TMT-set, purified using HiPPR Detergent Removal Resin and Pierce peptide desalting spin columns (both Thermo Scientific) and fractionated by basic reversed-phase chromatography on a XBridge BEH C18 column (3.5 µm, 2.1x250 mm, Waters). Peptides were separated using a stepped gradient from 3% to 67% acetonitrile (ACN) in 25 mM ammonia over 90 min at a flow rate of 200 µL/min, combined into 36 fractions, dried, and reconstituted in 3% ACN and 0.1% trifluoroacetic.

Fractions were analyzed on an Orbitrap Lumos Tribrid mass spectrometer equipped with a FAIMS Pro ion mobility interface and coupled to an Easy-nLC1200 system (all Thermo Fisher Scientific). Peptides were trapped on an Acclaim PepMap 100 C18 trap column (100 µm × 2 cm, 5 µm) and separated on an in-house packed analytical column (35 cm × 75 µm, 3 µm, Reprosil-Pur C18) using a stepped gradient from 5% to 80% ACN in 0.2% formic acid over 85 min at 300 nL/min. FAIMS Pro alternated between compensation voltages of −50 and −70, with identical data-dependent acquisition settings.

Full MS spectra were acquired at 120,000 resolutions over an m/z range of 375–1500. The most abundant precursors (charge 2–7) were selected within a 1.5 s cycle time using a 0.7 m/z isolation window and fragmented by CID (35%), with MS2 spectra acquired in the ion trap (rapid scan rate) and a dynamic exclusion of 60 s. The ten most intense MS2 fragment ions were selected for multi-notch MS3 analysis using HCD (55%), and MS3 spectra were recorded in the Orbitrap at 50,000 resolutions over an m/z range of 100–500.

Raw files were processed using Proteome Discoverer (v3.0, Thermo Fisher Scientific) and searched against the human SwissProt database (20,597 entries, February 2024) using

Sequest. Search parameters included a precursor tolerance of 5 ppm, fragment ion tolerance of 0.6 Da, and up to one missed cleavage. Methionine oxidation was set as a variable modification, while carbamidomethylation and TMTpro labeling of lysine residues and peptide N-termini were set as fixed modifications. PSM validation was performed using Percolator with a 1% FDR threshold. TMT reporter ions were quantified from MS3 HCD spectra with a 3 mmu tolerance and normalized to total peptide abundance. An SPS threshold of 65% and a Sequest HT score cutoff of 2 were applied. Only unique peptides were used for quantification, and proteins were required to meet a 1% FDR.

Transcriptomics

Biomarker Technologies (BMKGENE) performed RNA sequencing. Raw data (raw reads) of fastq format were first processed through in-house perl scripts. In this step, clean data (clean reads) were obtained by removing reads containing adapter or ploy-N, and low-quality reads from raw data. At the same time, Q20, Q30, GC-content and sequence duplication level of the clean data were calculated. All the downstream analyses were based on clean data with high quality.

String Tie (1.3.1) was used to calculate FPKMs (fragments per kilo-base of exon per million fragments mapped) of coding genes in each sample. Gene FPKMs were computed by summing the FPKMs of transcripts in each gene group. The resulting P-values were adjusted using Benjamini and Hochberg's approach for controlling the false discovery rate. Genes with an adjusted P <.01 and absolute value of log2(Fold change) >1 found by DESeq were assigned as differentially expressed.

Analysis of triglycerides and cholesteryl esters

The lipid method was adopted from a previous publication [3] with some modifications. Briefly: Extraction of lipids from the Spheroid pellets and from media was performed by an initial solubilization/homogenization step by adding 0.9% NaCl (aq): MeOH: Chloroform in the ratio [1:20:10] to give a total sample volume of 1 mL in each Eppendorf tube (saline was added first). The tubes were ultrasonicated for 10 minutes to facilitate homogenization. After homogenization 100 µl of sample (10%) was used for the liquid-liquid Bligh-Dyer extraction. The initial monophase solvent composition was [8:20:10] mL of 0.9%NaCl(aq):MeOH:Choloroform. The ratio was adjusted to [18:20:20] to induce phase separation, a lower organic phase and upper aqueous phase. The methanol solvent used during extraction contained 20 ng/ml stable isotope labeled triglycerides (TAG) and cholesteryl ester (CE) standard (EquiSPLASH SKU: 330731, Avanti Research). After the phase separation 1 ml of the lower organic layer was recovered into an LC-vial and evaporated to dryness under a stream of nitrogen. The remaining lipid film was solubilized in 500 µl organic solvent of acetonitrile/isopropanol having a ratio of [2:1], this was used as the injection solvent.

The samples were analyzed using Waters ACQUITY Premier liquid chromatography system with Waters HSS T3 2.1*100 mm column (SKU:186003539). Mobile phase A was methanol with 10 mM ammonium format and mobile phase B was isopropanol with 0.2% formic acid. A 19-minute gradient running between 5%-95% of mobile phase B at a flow of 0.3 mL/min was used. Data was acquired using a Waters Xevo TQ Absolute mass spectrometer detector using a multiple reaction monitoring method for the species expected to be most abundant in each class (CE=12 species and TAG=63 species). A low desolvation temperature of 200°C was used to prevent thermal degradation of the CE lipids. Building the acquisition method and peak integration of raw data was done using the software tools LipidCreator[2] and

Skyline (https://skyline.ms/). Quantification was done in the SAS statistical software and was based on the ratio of the analyte to internal standard area and amount of added internal standard added before the extraction. All TAG species were quantified using the TAG[15:0-18:1(d7)-15:0] internal standard and all CE lipids were quantified using the CE[18:1(d7)] internal standard. Amount of lipids per class was determined by summarizing the individual species. The use of a single internal standard for all lipid species in a class is a limitation for accurate absolute quantification. However, the relative amount can be determined with good linearity and precision which is sufficient for comparing samples run with the same method.

Data analysis

For transcriptomic analysis, differential expression analysis was performed using the DESeq R package (v1.10.1). The resulting P-values were adjusted using Benjamini and Hochberg's approach for controlling FDR. Genes with an adjusted P <.01 and log2(Fold change) >1 found by DESeq were assigned as differentially expressed.

For proteomic quantitative analysis, paired t-test was performed to get the P-values. Due to high biological variability and a small sample size, applying false discovery rate (FDR) was overly stringent, and no proteins passed this threshold. Therefore, proteins with P <.05 were considered significant [4].

Genes and proteins involved in different pathways were selected either from published literature or from the list of genes associated with each pathway in the enrichR databases [5-7]. For the integrative analysis of the transcriptomics and proteomics data, all the significant genes (FDR P <.01 and log2FC>1) and proteins (P <.05) were considered for analysis.

Heatmaps were generated based on protein expression data, with corresponding gene expression values arranged in the same order to facilitate interpretation. A scatter plot was used to visualize the integrative analysis, comparing the transcriptomic Vs. proteomic expression changes following incubation with FA+TGFB1. Log2FC between regular media ("No FA + No TGFB1") and "FA + TGFB1" conditions were calculated and plotted.

Liver tissue gene expression data was obtained from the GepLiver database [8], which stratifies samples into four fibrosis categories: no fibrosis, low fibrosis, high fibrosis, and cirrhosis. Statistical significance was assessed using Student’s unpaired t-test. Schematic representation figures were created using Biorender.com.

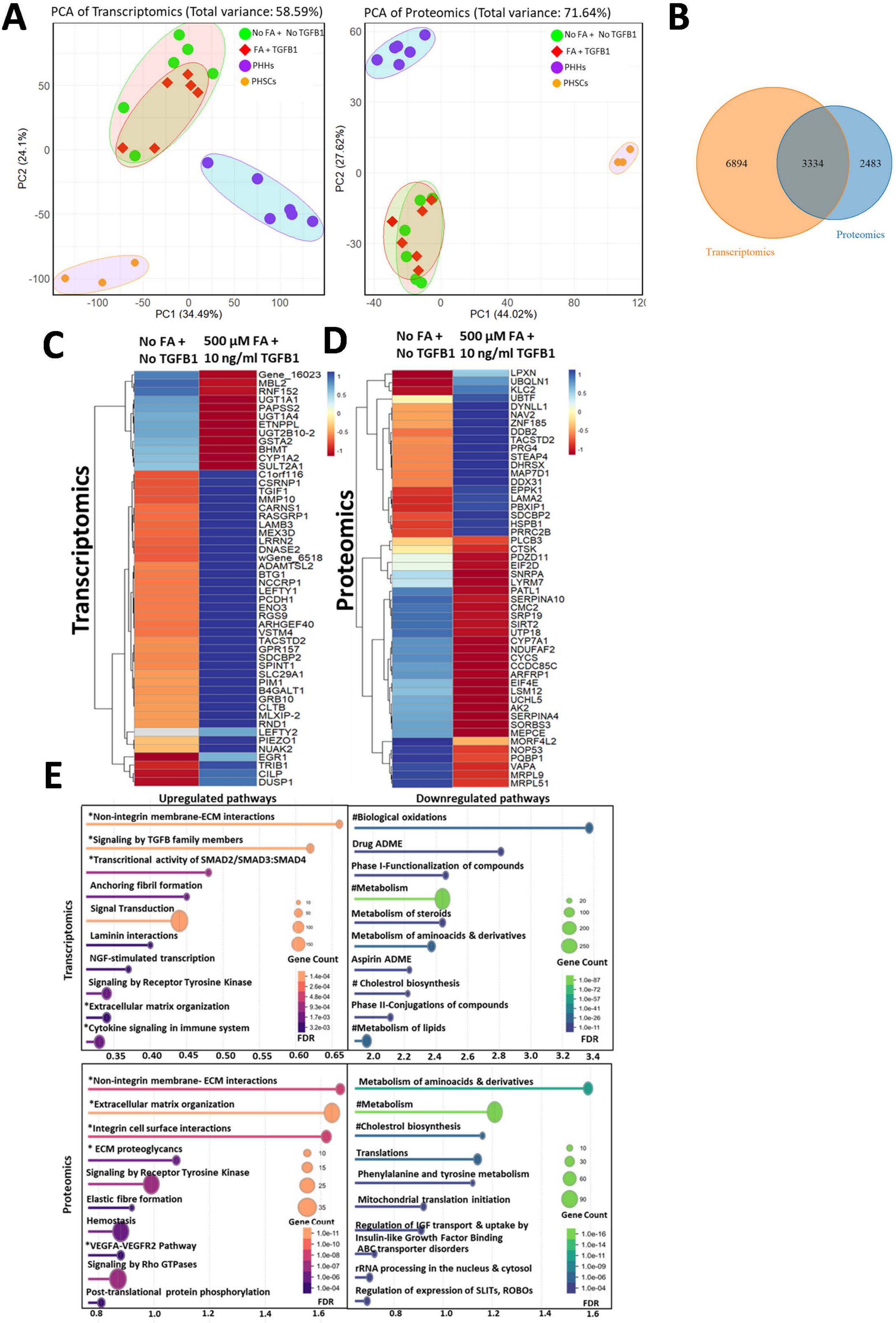
A
PCA of Transcriptomics (Total variance: 58.59%)
No FA + No TGFB1
FA + TGFB1
PHHs
PHSCs
PC2 (24.1%)
PC1 (34.49%)
PCA of Proteomics (Total variance: 71.64%)
PC2 (27.62%)
PC1 (44.02%)
B
6894
3334
2483
Transcriptomics
Proteomics
C
No FA + No TGFB1
500 µM FA + 10 ng/ml TGFB1
Transcriptomics
Gene_16023
MBL2
RNF152
UGT1A1
PAPSS2
UGT1A4
ETNPPL
UGT2B10-2
GSTA2
BHMT
CYP1A2
SULT2A1
C1orf116
CSRNP1
TGIF1
MMP10
CARNS1
RASGRP1
LAMB3
MEX3D
LRRN2
DNASE2
wGene_6518
ADAMTSL2
BTG1
NCCRP1
LEFTY1
PCDH1
ENO3
RGS9
ARHGEF40
VSTM4
TACSTD2
GPR157
SDCBP2
SPINT1
SLC29A1
PIM1
B4GALT1
GRB10
CLTB
MLXIP-2
RND1
LEFTY2
PIEZO1
NUAK2
EGR1
TRIB1
CILP
DUSP1
D
No FA + No TGFB1
500 µM FA + 10 ng/ml TGFB1
Proteomics
LPXN
UBQLN1
KLC2
UBTF
DYNLL1
NAV2
ZNF185
DDB2
TACSTD2
PRG4
STEAP4
DHRSX
MAP7D1
DDX31
EPPK1
LAMA2
PBXIP1
SDCBP2
HSPB1
PRRC2B
PLCB3
CTSK
PDZD11
EIF2D
SNRPA
LYRM7
PATL1
SERPINA10
CMC2
SRP19
SIRT2
UTP18
CYP7A1
NDUFAF2
CYCS
CCDC85C
ARFRP1
EIF4E
LSM12
UCHL5
AK2
SERPINA4
SORBS3
MEPCE
MORF4L2
NOP53
PQBP1
VAPA
MRPL9
MRPL51
E
Upregulated pathways
Downregulated pathways
Transcriptomics
*Non-integrin membrane-ECM interactions
*Signaling by TGFB family members
*Transcritional activity of SMAD2/SMAD3:SMAD4
Anchoring fibril formation
Signal Transduction
Laminin interactions
NGF-stimulated transcription
Signaling by Receptor Tyrosine Kinase
*Extracellular matrix organization
*Cytokine signaling in immune system
Gene Count
FDR
#Biological oxidations
Drug ADME
Phase I-Functionalization of compounds
#Metabolism
Metabolism of steroids
Metabolism of aminoacids & derivatives
Aspirin ADME
Cholestrol biosynthesis
Phase II-Conjugations of compounds
#Metabolism of lipids
Gene Count
FDR
Proteomics
*Non-integrin membrane- ECM interactions
*Extracellular matrix organization
*Integrin cell surface interactions
* ECM proteoglycancs
Signaling by Receptor Tyrosine Kinase
Elastic fibre formation
Hemostasis
*VEGFA-VEGFR2 Pathway
Signaling by Rho GTPases
Post-translational protein phosphorylation
Gene Count
FDR
Metabolism of aminoacids & derivatives
#Metabolism
#Cholestrol biosynthesis
Translations
Phenylalanine and tyrosine metabolism
Mitochondrial translation initiation
Regulation of IGF transport & uptake by Insulin-like Growth Factor Binding
ABC transporter disorders
rRNA processing in the nucleus & cytosol
Regulation of expression of SLITs, ROBOs
Gene Count
FDR

Figure S1: Overall distribution and analysis of proteomic and transcriptomic data. Principal component analysis (PCA) of (A) transcriptomic and proteomic data. All the genes or proteins detected using RNA-seq or LC-MS were used to generate the PCA. Six donors for PHHs, three donors for PHSCs, cultured in 2D, as well as the di-lineage spheroids cultured in regular media or with 500 µM FA+ 10ng/ml TGFB1 were processed for proteomics and transcriptomics. Plots were generated using ggplot2 R package. (B) Venn diagram depicting the common genes and protein detected overall in the dataset. The top 50 differentially expressed (C) genes and (D) proteins identified in spheroids incubated with di-lineage hepatic spheroids with 500 µM FA (OA+PA, 2:1) and TGFB1 (10 ng/ml), compared to regular media (no FA + no TGFB1). Data are expressed as median of log2FC values (log2TPM values for transcriptomics and log2-relative protein abundance for proteomics) of 6 donors with or without FA and TGFB1 incubation. (E) Top 10 upregulated and downregulated pathways as enriched in Reactome database (curated from STRING database[9]) using all the significant genes/proteins (detected by transcriptomics or proteomics) either upregulated or downregulated. * and # denotes pathways associated with extracellular matrix and metabolic pathways, respectively. X-axis represents the signal (scores) of the pathways.

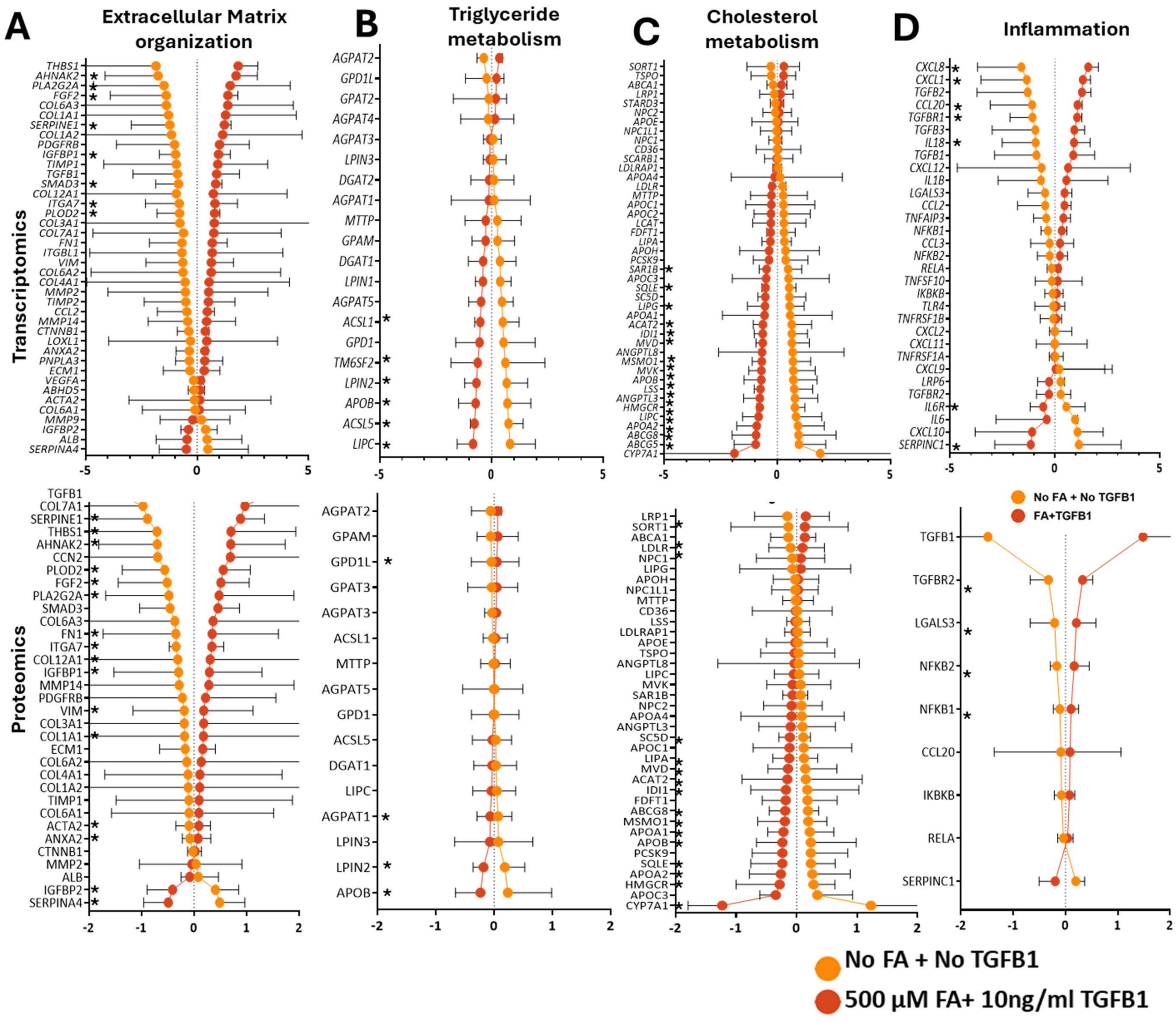

A
Extracellular Matrix organization
B
Triglyceride metabolism
C
Cholesterol metabolism
D
Inflammation
Transcriptomics
Proteomics
THBS1 AHNAK2 PLA2G2A FGF2 COL6A3 COL1A1 SERPINE1 COL1A2 PDGFRB IGFBP1 TIMP1 TGFB1 SMAD3 COL12A1 ITGA7 PLOD2 COL3A1 COL7A1 FN1 ITGBL1 VIM COL6A2 COL4A1 MMP2 TIMP2 CCL2 MMP14 CTNNB1 LOXL1 ANXA2 PNPLA3 ECM1 VEGFA ABHD5 ACTA2 COL6A1 MMP9 IGFBP2 ALB SERPINA4
AGPAT2 GPD1L GPAT2 AGPAT4 AGPAT3 LPIN3 DGAT2 AGPAT1 MTTP GPAM DGAT1 LPIN1 AGPAT5 ACSL1 GPD1 TM6SF2 LPIN2 APOB ACSL5 LIPC
SORT1 TSPO ABCA1 LRP1 STARD3 NPC2 APOE NPC1L1 NPC1 CD36 SCARB1 LDLRAP1 APOA4 LDLR MTTP APOC1 APOC2 LCAT FDFT1 LIPA APOH PCSK9 SAR1B APOC3 SQLE SC5D LIPG APOA1 ACAT2 IDI1 MVD ANGPTL8 MSMO1 MVK APOB LSS ANGPTL3 HMGCR LIPC APOA2 ABCG8 ABCG5 CYP7A1
CXCL8 CXCL1 TGFB2 CCL20 TGFBR1 TGFB3 IL18 TGFB1 CXCL12 IL1B LGALS3 CCL2 TNFAIP3 NFKB1 CCL3 NFKB2 RELA TNFSF10 IKBKB TLR4 TNFRSF1B CXCL2 CXCL11 TNFRSF1A CXCL9 LRP6 TGFBR2 IL6R IL6 CXCL10 SERPINC1
-5 0 5
TGFB1 COL7A1 SERPINE1 THBS1 AHNAK2 CCN2 PLOD2 FGF2 PLA2G2A SMAD3 COL6A3 FN1 ITGA7 COL12A1 IGFBP1 MMP14 PDGFRB VIM COL3A1 COL1A1 ECM1 COL6A2 COL4A1 COL1A2 TIMP1 COL6A1 ACTA2 ANXA2 CTNNB1 MMP2 ALB IGFBP2 SERPINA4
AGPAT2 GPAM GPD1L GPAT3 AGPAT3 ACSL1 MTTP AGPAT5 GPD1 ACSL5 DGAT1 LIPC AGPAT1 LPIN3 LPIN2 APOB
LRP1 SORT1 ABCA1 LDLR NPC1 LIPG APOH NPC1L1 MTTP CD36 LSS LDLRAP1 APOE TSPO ANGPTL8 LIPC MVK SAR1B NPC2 APOA4 ANGPTL3 SC5D APOC1 LIPA MVD ACAT2 IDI1 FDFT1 ABCG8 MSMO1 APOA1 APOB PCSK9 SQLE APOA2 HMGCR APOC3 CYP7A1
No FA + No TGFB1
FA+TGFB1
TGFB1 TGFBR2 LGALS3 NFKB2 NFKB1 CCL20 IKBKB RELA SERPINC1
-2 -1 0 1 2
No FA + No TGFB1
500 µM FA+ 10ng/ml TGFB1

Figure S2: Genes/proteins related to extracellular matrix, triglyceride, cholesterol metabolism, and inflammation are altered in FA+TGFB1 incubated di-lineage spheroids. Specific genes involved in different pathways related to MASH are plotted from Transcriptomic and proteomic data of di-lineage spheroids incubated with or without FA+TGFB1. (A) extracellular matrix organization, (B) triglyceride metabolism, (C) cholesterol metabolism and (D) inflammation related genes and proteins shown as mean ± SD of log2FC of expression (log2TPM for transcriptomics) and log2(relative protein abundance) for proteomics). P-values calculated using DeSeq2 for transcriptome or paired t-test for proteome data. *P <.05

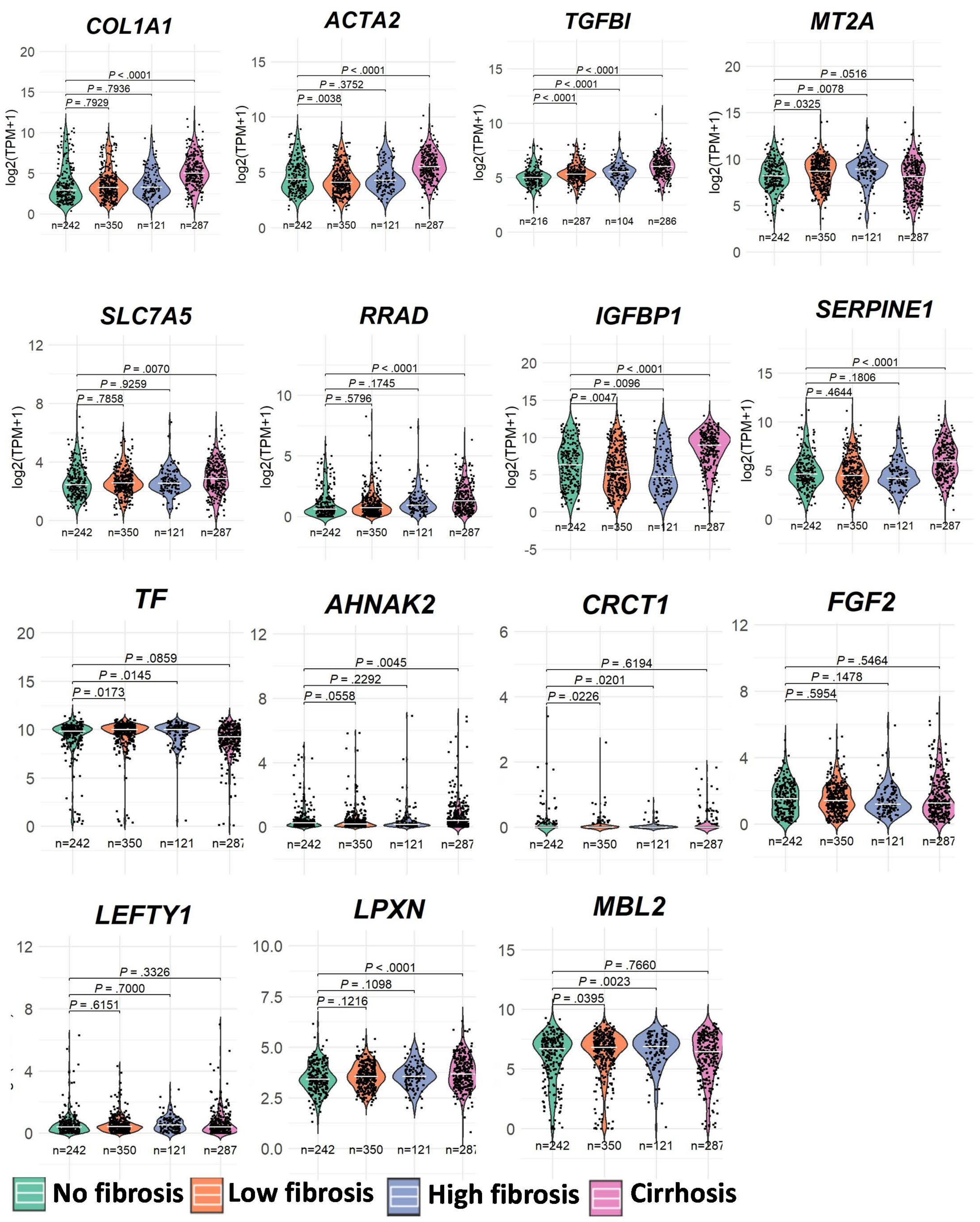

COL1A1
P < .0001
P = .7936
P = .7929
n=242 n=350 n=121 n=287
ACTA2
P < .0001
P = .3752
P = .0038
n=242 n=350 n=121 n=287
TGFBI
P < .0001
P < .0001
P < .0001
n=216 n=287 n=104 n=286
MT2A
P = .0516
P = .0078
P = .0325
n=242 n=350 n=121 n=287
SLC7A5
P = .0070
P = .9259
P = .7858
n=242 n=350 n=121 n=287
RRAD
P < .0001
P = .1745
P = .5796
n=242 n=350 n=121 n=287
IGFBP1
P < .0001
P = .0096
P = .0047
n=242 n=350 n=121 n=287
SERPINE1
P < .0001
P = .1806
P = .4644
n=242 n=350 n=121 n=287
TF
P = .0859
P = .0145
P = .0173
n=242 n=350 n=121 n=287
AHNAK2
P = .0045
P = .2292
P = .0558
n=242 n=350 n=121 n=287
CRCT1
P = .6194
P = .0201
P = .0226
n=242 n=350 n=121 n=287
FGF2
P = .5464
P = .1478
P = .5954
n=242 n=350 n=121 n=287
LEFTY1
P = .3326
P = .7000
P = .6151
n=242 n=350 n=121 n=287
LPXN
P < .0001
P = .1098
P = .1216
n=242 n=350 n=121 n=287
MBL2
P = .7660
P = .0023
P = .0395
n=242 n=350 n=121 n=287
log2(TPM+1)
No fibrosis
Low fibrosis
High fibrosis
Cirrhosis

Figure S3: Comparison of the integrated omics results with data from liver gene expression atlas. A comparison between individuals without fibrosis (No fibrosis) vs. those with different stages of fibrosis (low, high and cirrhosis) in the GepLiver database for different genes as reported at top of each panel. The number of individuals for each group is reported in the plot. P-values were calculated using unpaired Student's t-test.

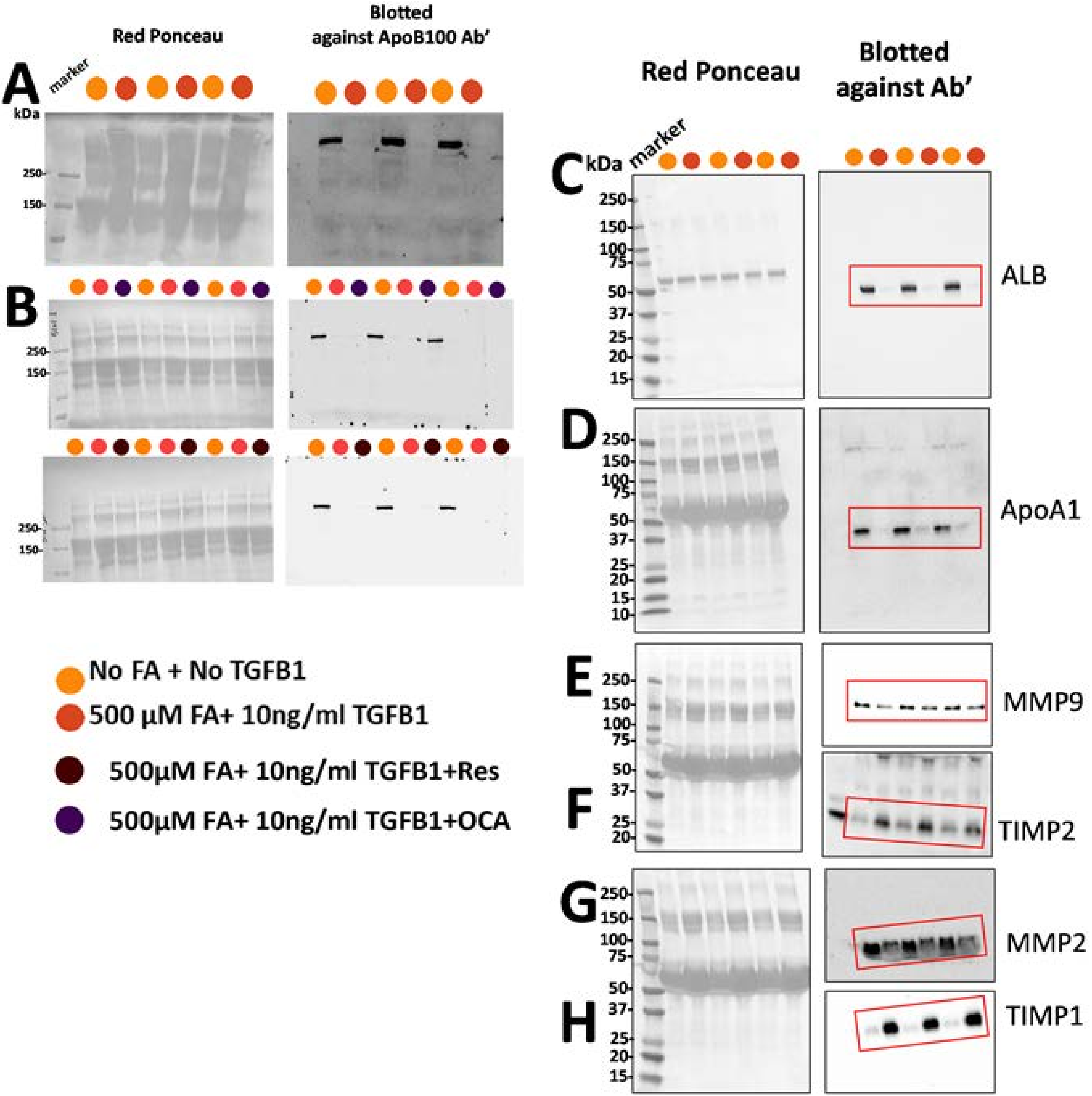

Red Ponceau
Blotted against ApoB100 Ab'
A
marker
kDa
250
150
B
250
150
250
150
No FA + No TGFB1
500 μM FA+ 10ng/ml TGFB1
500μM FA+ 10ng/ml TGFB1+Res
500μM FA+ 10ng/ml TGFB1+OCA
Red Ponceau
Blotted against Ab'
C
kDa
marker
250
150
100
75
50
37
25
20
15
ALB
D
250
150
100
75
50
37
25
20
15
10
ApoA1
E
250
150
100
75
50
37
25
20
MMP9
F
TIMP2
G
250
150
100
75
50
37
25
20
15
MMP2
H
TIMP1

Figure S4: Original Western blot images and corresponding red ponceau images used for protein normalization. Panel A corresponds to figure 1E; panel B corresponds to 2E. Panel C and D corresponds to figure 1G; E-H corresponds to figure 2H. Red marked areas are shown in the main figures.

**Donors**	**Cell types**	**Sex**	**Age**	**Race**	**BMI**	**spheroids combination**
AMC	PHH	F	26	Caucasian	17.6	1
JEL	PHH	F	27	African American	28.2	2
IIN	PHH	F	57	Caucasian	21.3	3
INM	PHH	M	56	Caucasian	22.4	4
OMA	PHH	M	37	Caucasian	24.8	5
BXU	PHH	F	6	Caucasian	22.6	6
XGD	PHSC	F	45	Caucasian	28.1	1
TFE	PHSC	M	50	Caucasian	20.4	2
LVC	PHSC	F	63	Caucasian	39.1	3
BFL	PHSC	F	53	Caucasian	24.5	4
CHM2104-SC-P3-2-Z	PHSC	M	68	Caucasian	25.06	5
CyHuf19006-SC-P2-Z	PHSC	F	65	Caucasian	26.56	6
CyHuf20001-SC-P1-Z	PHSC	F	55	Caucasian	25.2	NA

Supplementary Table 1: Characteristics of primary human hepatocyte and primary hepatic stellate cells used in this study.

References:

1. Sasidharan, K., et al., *IL32 downregulation lowers triglycerides and type I collagen in di-lineage human primary liver organoids.* Cell Rep Med, 2024. **5**(1): p. 101352.
2. Mancina, R.M., et al., *PSD3 downregulation confers protection against fatty liver disease.* Nat Metab, 2022. **4**(1): p. 60-75.
3. Ciociola, E., et al., *Downregulation of the MARC1 p.A165 risk allele reduces hepatocyte lipid content by increasing beta-oxidation.* Clin Mol Hepatol, 2025. **31**(2): p. 445-459.
4. Levin, Y., *The role of statistical power analysis in quantitative proteomics.* Proteomics, 2011. **11**(12): p. 2565-2567.
5. Chen, E.Y., et al., *Enrichr: interactive and collaborative HTML5 gene list enrichment analysis tool.* BMC bioinformatics, 2013. **14**: p. 1-14.
6. Kuleshov, M.V., et al., *Enrichr: a comprehensive gene set enrichment analysis web server 2016 update.* Nucleic acids research, 2016. **44**(W1): p. W90-W97.
7. Xie, Z., et al., *Gene set knowledge discovery with Enrichr.* Current protocols, 2021. **1**(3): p. e90.
8. Li, Z., et al., *GepLiver: an integrative liver expression atlas spanning developmental stages and liver disease phases.* Scientific Data, 2023. **10**(1): p. 376.
9. Szklarczyk, D., et al., *The STRING database in 2023: protein–protein association networks and functional enrichment analyses for any sequenced genome of interest.* Nucleic acids research, 2023. **51**(D1): p. D638-D646.
